# *Drosophila* anti-nematode and antibacterial immune regulators revealed by RNA-Seq

**DOI:** 10.1186/s12864-015-1690-2

**Published:** 2015-07-11

**Authors:** Julio C. Castillo, Todd Creasy, Priti Kumari, Amol Shetty, Upasana Shokal, Luke J. Tallon, Ioannis Eleftherianos

**Affiliations:** Insect Infection and Immunity Lab, Department of Biological Sciences, Institute for Biomedical Sciences, The George Washington University, Washington DC, 20052 USA; Laboratory of Malaria and Vector Research, National Institutes of Health, Rockville, MD 20852 USA; Institute for Genome Sciences, Department of Microbiology and Immunology, University of Maryland School of Medicine, Baltimore, MD 21201 USA

**Keywords:** *Drosophila*, *Photorhabdus*, *Heterorhabditis*, RNA-sequencing, Transcriptomics, Immunity, Infection, Parasitism

## Abstract

**Background:**

*Drosophila melanogaster* activates a variety of immune responses against microbial infections. However, information on the *Drosophila* immune response to entomopathogenic nematode infections is currently limited. The nematode *Heterorhabditis bacteriophora* is an insect parasite that forms a mutualistic relationship with the gram-negative bacteria *Photorhabdus luminescens*. Following infection, the nematodes release the bacteria that quickly multiply within the insect and produce several toxins that eventually kill the host. Although we currently know that the insect immune system interacts with *Photorhabdus,* information on interaction with the nematode vector is scarce.

**Results:**

Here we have used next generation RNA-sequencing to analyze the transcriptional profile of wild-type adult flies infected by axenic *Heterorhabditis* nematodes (lacking *Photorhabdus* bacteria), symbiotic *Heterorhabditis* nematodes (carrying *Photorhabdus* bacteria), and *Photorhabdus* bacteria alone. We have obtained approximately 54 million reads from the different infection treatments. Bioinformatic analysis shows that infection with *Photorhabdus* alters the transcription of a large number of *Drosophila* genes involved in translational repression as well in response to stress. However, *Heterorhabditis* infection alters the transcription of several genes that participate in lipidhomeostasis and metabolism, stress responses, DNA/protein sythesis and neuronal functions. We have also identified genes in the fly with potential roles in nematode recognition, anti-nematode activity and nociception.

**Conclusions:**

These findings provide fundamental information on the molecular events that take place in *Drosophila* upon infection with the two pathogens, either separately or together. Such large-scale transcriptomic analyses set the stage for future functional studies aimed at identifying the exact role of key factors in the *Drosophila* immune response against nematode-bacteria complexes.

**Electronic supplementary material:**

The online version of this article (doi:10.1186/s12864-015-1690-2) contains supplementary material, which is available to authorized users.

## Background

Host innate immune responses are broadly conserved across many phyla [1]. The study of the interaction between invertebrate model hosts and pathogenic organisms provides insights into the mechanisms underlying pathogen virulence and host immunity, and complements the use of mammalian models by enabling whole-animal high throughput infection assays and genome wide transcriptome analyses [2]. Despite impressive advances in the broad field of innate immunity, our understanding of the molecules that participate in the host immune response to nematode infections remains incomplete [3]. Novel anti-nematode immune responses in the host are likely to be identified in model systems in which the host has a sequenced genome and can be genetically manipulated. The common fruit fly, *Drosophila melanogaster,* with a vast number of genetics and genomics tools available, is widely recognized as an outstanding model to analyze immune signaling pathways and elucidate the molecular and genetic basis of immune defense mechanisms [4–6].

The insect pathogenic nematode *Heterorhabditis bacteriophora* is emerging as a promising parasitic organism for studying nematode pathogenicity and characterizing the function of novel host factors that contribute to anti-nematode immune reactions [7, 8]. *Heterorhabditis* nematodes form a mutually beneficial symbiotic relationship with the Gram-negative bacteria of the *Enterobacteriaceae* family, *Photorhabdus luminescens*, which are found in the gut of the worms [9]. *Heterorhabditis* infective juvenile (IJ) worms belong to an obligate stage in the nematode life cycle that is required for infection of the insect. This stage is analogous to the *Caenorhabditis elegans* dauer stage and the developmentally arrested infective third stage larva (L3) of many mammalian parasitic nematodes [10]. IJs gain entry to the insect through natural openings or by penetrating the cuticle. Once inside, the IJ resume development and expel *Photorhabdus* into the hemolymph where the bacteria begin to divide. After 2-3 days of bacterial growth the insect succumbs to the infection with the concomitant conversion of the internal organs and tissues into bacterial biomass, facilitated by a wide range of toxins, virulence factors and hydrolytic enzymes produced by the bacteria [11, 12]. For two to three generations the developing nematodes feed on the bacterial biomass until the insect carcass is consumed, whereupon adult development is suppressed and the IJ stage accumulates. These non-feeding IJ containing their mutualistic bacteria emerge into the soil to seek new hosts [13]. We and others have previously shown that *Heterorhabditis* is a potent pathogen of *Drosophila,* and have begun using the *Drosophila-Heterorhabditis* model system to understand the molecular interplay between insect immune function and nematode parasitic strategies [14–18].

Whole genome mRNA sequencing (RNA-Seq) technologies have been a significant advance for high-throughput transcriptome analyses, as they can generate hundreds of millions reads in a single sequencing run [19, 20]. RNA-Seq is more sensitive, quantitative and efficient, and it has higher reproducibility compared to previously used hybridization-based microarray techniques [21]. RNA-Seq has already produced exciting and novel information in the study of various diseases [22, 23]. This powerful tool is becoming increasingly attractive for investigating the transcriptional profiles in model and non-model organisms [24, 25]. Recent works have started to report the use of RNA-Seq (Illumina or 454-pyrosequencing) for the comprehensive understanding of the transcriptional regulation of genes that participate in pathogen virulence and host innate immune processes [26].

Here we have infected *Drosophila melanogaster* adult flies with symbiotic *Heterorhabditis* (nematodes carrying *Photorhabdus*), axenic *Heterorhabditis* (nematodes lacking *Photorhabdus*), and *Photorhabdus* bacteria alone and used RNA-Seq to analyze the transcriptional response of flies to the pathogens, either separately or together. Our goal was to identify the number and nature of *Drosophila* genes that are differentially regulated upon infection with the nematodes and their associated bacteria. We find that distinct types of genes are regulated during infection of the fly by each of the two pathogens. Therefore these results indicate that different sets of genes are involved in the interaction between the fly and the nematodes or their bacteria, and that the fly employs distinct strategies to fight infection against *Heterorhabditis* nematode parasites and their mutualistic *Photorhabdus* bacteria.

## Results

### *Heterorhabditis* nematodes and *Photorhabdus* bacteria produce distinct transcriptomic profiles in *Drosophila*

**Fig. 1:**
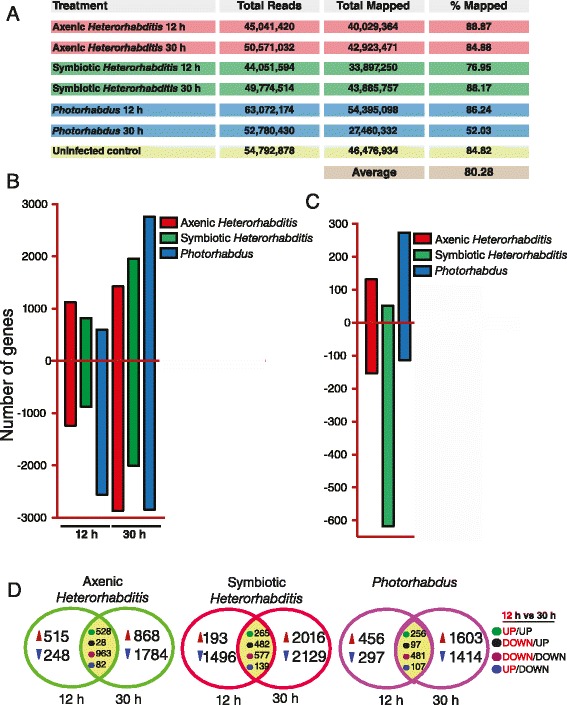
Infection of adult flies with *Heterorhabditis* nematodes or their *Photorhabdus* bacteria elicits distinct transcriptomic profiles. **a** Transcriptome summary (number of reads and percentage mapped to the *D. melanogaster* genome) from flies infected by *Heterorhabditis* axenic or symbiotic nematodes, or *Photorhabdus* bacteria at 12 and 30 h post-infection. **b** Differential gene transcription (upregulated/downregulated genes) in flies at 12 h and 30 h post-infection with *Heterorhabditis* axenic or symbiotic nematodes, or *Photorhabdus* bacteria alone. **c** CUFFLINKS analysis of differentially expressed transcripts between the 12 and 30 h time-points in flies infected by *Heterorhabditis* axenic or symbiotic nematodes, or *Photorhabdus* bacteria alone. **d** Venn diagrams showing the number of *Drosophila* genes that are differentially expressed (upregulated or downregulated) at 12 h only or at 30 h only or at both time-points after infection with *Heterorhabditis* axenic or symbiotic nematodes, or their *Photorhabdus* bacteria alone. Expression patterns are indicated (UP/UP: gene upregulation at both 12 and 30 h, DOWN/UP: gene downregulation at 12 h and upregulation at 30 h, DOWN/DOWN: gene downregulation at both time-points, UP/DOWN: gene upregulation at 12 h and downregulation at 30 h)

We generated complete transcriptomes from *Drosophila* wild-type adult flies infected by the insect pathogenic nematodes *Heterorhabditis* and their mutualistic bacteria *Photorhabdus*, separately or together. We examined gene transcription for two time-points, 12 and 30 h post-infection with the pathogens (Fig. 1). These time-points correspond to the initial entry and spread of the pathogens in the fly (12 h post-infection) and to the establishment of disease (30 h post-infection) [27]. The numbers of sequence reads mapped to 80.28 % of the *D. melanogaster* genome (Fig. 1a). Similarly, the high number of reads sequenced had more than 90 % coverage of the *D. melanogaster* genome (Additional file 1: Figure S1). The quantitative real-time RT-PCR (qRT-PCR) analysis of randomly selected genes (*CG34040*, *CG64267*, *CG9468*, *CG11909*, *CG6524*, *CG17571*, *CG10374*) using gene-specific primers (Additional file 1: Table S1) validated the RNA-Seq data (Additional file 1: Figure S2). We found upregulation for *CG34040*, *CG64267*, *CG11909* and downregulation for *CG9468*, *CG6524*, *CG17571*, *CG10374* although the level of transcription detected was higher by RNA-Seq with the exception of the gene *CG11909*.

The highest number of differentially expressed genes was observed in flies infected by *Photorhabdus* at 30 h post-infection (Fig. 1b). Strikingly, we found that the vast majority of fly genes (82 %, 2555 genes) were downregulated at 12 h post-infection with *Photorhabdus,* and similar numbers of genes were upregulated or downregulated at 30 h after infection with the bacteria (2763 and 2845 genes, respectively). At 12 h post-infection with axenic *Heterorhabditis*, there were 1125 upregulated genes (47 %) and 1238 downregulated genes (53 %). Similarly, infection with symbiotic nematodes upregulated 819 genes (48 %) and downregulated 871 genes (52 %). We also found that axenic nematodes at 30 h post-infection downregulated a higher number of fly genes (67 %, 2868 genes) compared to those downregulated by symbiotic worms (51 %, 2002 genes) (Fig. 1b).

To identify the numbers of differentially expressed gene isoforms induced by each pathogen at each time-point, we performed Cufflinks analysis [28]. We found 273 gene isoforms upregulated by *Photorhabdus*, whereas infection by axenic or symbiotic nematodes upregulated fewer gene isoforms in the fly (131 and 52, respectively) (Fig. 1c). However, the largest number of downregulated gene isoforms was found in flies infected by symbiotic or axenic worms (618 and 154, respectively) and fewer (114) in flies infected by *Photorhabdus* (Fig. 1c).

To determine the number of genes that are transcriptionally regulated upon infection with *Heterorhabditis* and *Photorhabdus*, we performed pairwise multiple comparison analyses. We found that the number of differentially regulated genes at 12 and 30 h post-infection varied among the different types of infection (Fig. 1d). We focused on the group of genes that were regulated at both time-points and observed: (i) several genes that were downregulated at 12 h post-infection and upregulated at 30 h post-infection (28 genes in flies infected by axenic worms, 482 in flies infected by symbiotic worms and 97 in flies infected by the bacteria); ii) a higher number of upregulated genes in flies infected by axenic worms (528 genes) compared to those upregulated by symbiotic worms (265 genes) and the bacteria alone (256 genes); iii) a smaller number of genes that were upregulated at 12 h and downregulated at 30 h by infection with axenic nematodes, symbiotic nematodes, or *Photorhabdus* alone (82, 139 and 107 genes, respectively; (iv) a large number of genes that remained transcriptionally downregulated upon infection with symbiotic worms, axenic worms or the bacteria alone (963, 577, and 481, respectively). These results suggest that a large set of genes is differentially regulated in *Drosophila* adult flies during the early and late stages of infection by *Heterorhabditis* nematodes and their mutualistic *Photorhabdus* bacteria.

### *Heterorhabditis* and *Photorhabdus* infection leads to changes in specific molecular pathways and biological activities in *Drosophila*

**Fig. 2:**
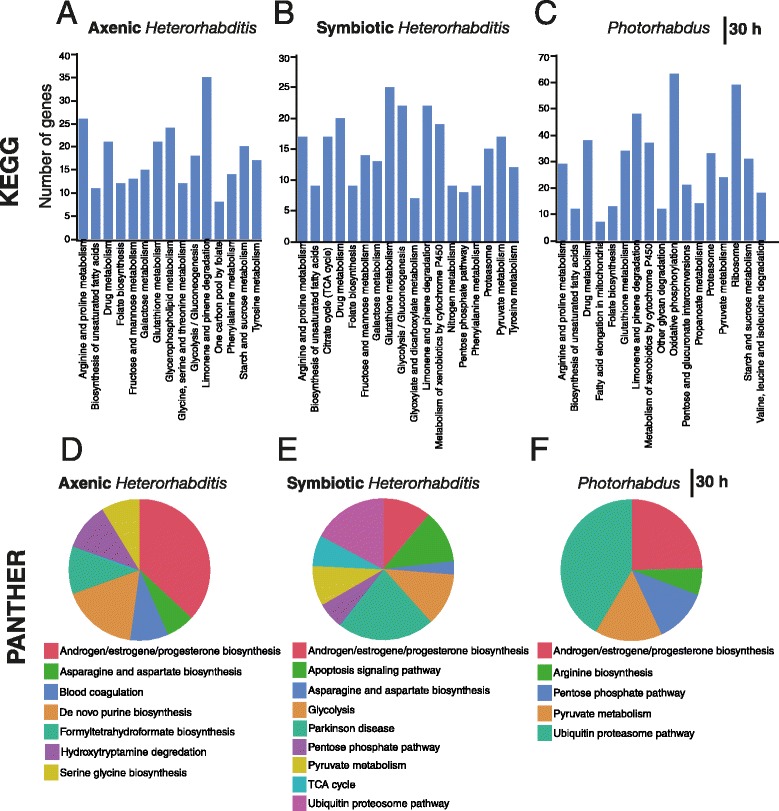
Infection of adult flies with *Heterorhabditis* nematodes or their *Photorhabdus* bacteria induces diverse physiological responses. Representative KEGG pathway categories in flies infected by **a** *Heterorhabditis* axenic nematodes, **b** *Heterorhabditis* symbiotic nematodes, or **c** *Photorhabdus* bacteria at 30 h post-infection. The number of genes represents those that were only found associated with a particular pathway. Representative PANTHER pathway categories in flies infected by **d** *Heterorhabditis* axenic nematodes, **e** *Heterorhabditis* symbiotic nematodes, or **f** *Photorhabdus* bacteria at 30 h post-infection

To identify the molecular pathways and biological activities regulated by the nematodes and their bacteria, we performed Database for Annotation, Visualization and Integrated Discovery (DAVID) analysis by interrogating the Kyoto Encyclopedia of Genes and Genomes (KEGG) and Protein ANalysis THrough Evolutionary Relationships (PANTHER) databases [29–32] (Fig. 2). At 12 h after infection with axenic or symbiotic nematodes, there was a significant change in the transcription of a large set of genes, which generated distinct pathway categories (KEGG). For instance, we found that axenic and symbiotic nematodes elicited the enrichment of genes involved in metabolism of xenobiotics by cytochrome P450 and glycolysis, while symbiotic nematodes elicited the enrichment of genes involved in lysosome function and apoptosis signaling (Additional file 1: Figure S3; Additional file 2: Dataset S1).

We also found that a large number of genes within certain pathways (such as limonene and pinene, tyrosine degradation, arginine and proline metabolism, biosynthesis of unsaturated fatty acids, drug metabolism, folate biosynthesis, glutathione metabolism and glycosylation) were transcriptionally altered following infection of flies with the nematodes and their bacteria (together or alone) for 30 h (Fig. 2a,b,c). We further found downregulation of ornithine decarboxylase related genes in all three types of infections, which might suggest the induction of anti-inflammatory responses in the fly (Additional file 2: Dataset S1). There was also strong downregulation of a large number of genes in the ubiquitin/proteasome degradation pathways in *Photorhabdus* infected flies, but not in flies infected by axenic or symbiotic nematodes (Additional file 2: Dataset S1).

PANTHER analysis pointed towards pathways that were not identified by KEGG analysis (Fig. 2d,e,f). Infection of flies with the two pathogens for 30 h affected the androgen/estrogen/progesterone biosynthesis pathway, which includes genes that take part in lipid metabolism, steroid hormone metabolism, and cholesterol metabolism. Infection with axenic or symbiotic nematodes caused the enrichment of genes involved in asparagine/aspartate biosynthesis. Infection with symbiotic worms or *Photorhabdus* bacteria alone induced the enrichment of genes involved in ubiquitin-proteosome degradation of proteins, pyruvate metabolism and pentose-phosphate pathway. Therefore pathway analyses reveal certain molecular/biological signatures in the fly and provide hints on the physiological events that take place during infection with the nematodes and their associated bacteria.

### *Heterorhabditis* and *Photorhabdus* infection regulates the transcription of a wide range of protein-coding genes in the fly genes

**Fig. 3:**
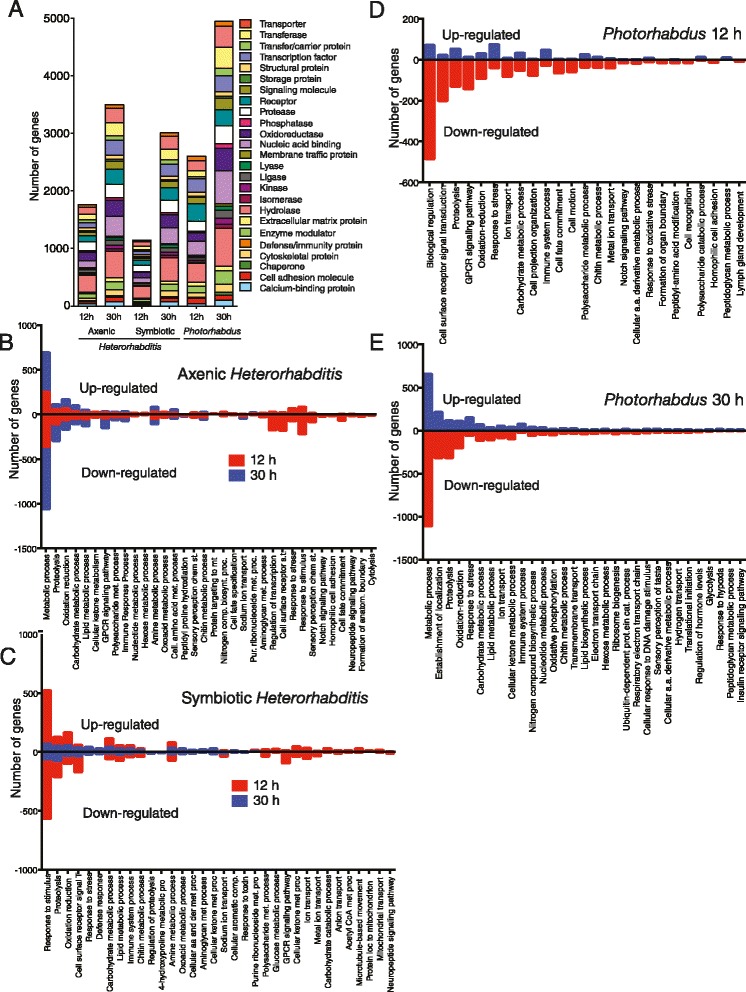
Infection of adult flies with *Heterorhabditis* or *Photorhabdus* trigger the expression of diverse proteins. **a** Representative protein-based Gene Ontology (GO) groups for genes differentially induced by *Heterorhabditis* axenic or symbiotic nematodes, or *Photorhabdus* bacteria alone at 12 and 30 h post-infection with the pathogens. Each bar represents a subset of the most representative non-redundant upregulated and downregulated genes. Numbers of upregulated and downregulated genes upon infection with *Heterorhabditis* axenic (**b**), symbiotic (**c**) nematodes, or *Photorhabdus* bacteria (**d**, **e**) at 12 h and 30 h post-infection with the pathogens. Each bar includes genes that fall into the same molecular function category. GO analysis was performed using the global list of differentially expressed genes for each infection type and time-point

To identify the major protein families and biological processes associated with the fly genes that are differentially regulated by *Heterorhabditis* and *Photorhabdus* infection, we conducted gene ontology (GO) analysis [33] (Fig. 3a). At late stages of infection with the pathogens there was a substantial increase in the number of protein-coding genes. For example, we found a dramatic increase in the expression of hydrolase genes upon infection with the nematodes and their bacteria. At 30 h post-infection with the pathogens the top 15 protein categories included hydrolases, nucleic acid binding proteins, oxido-reductases, transferases, transporters, proteases, transcription factors, receptors, enzyme modulators, signaling molecules, cytoskeletal proteins, ligases, transfer/carrier proteins, calcium-binding proteins and kinases.

GO analysis also showed that infection with axenic worms strongly downregulated a large number of genes associated with metabolic process at both 12 and 30 h post-infection (356 and 700 genes, respectively) (Fig. 3b). However, the number of similar genes that were upregulated upon infection with axenic worms increased from 252 to 442. Genes involved in proteolysis and G-protein coupled receptor (GPCR) signaling were strongly downregulated at 30 h, while all 7 genes associated with peptidyl hydroxylation were downregulated at 12 h post-infection. We further found that only 23 immunity-related genes were upregulated at 30 h and 9 genes at 12 h, while 29 genes involved in chitin metabolic process were downregulated at 30 h post-infection.

GO analysis further revealed that infection of flies for 30 h with symbiotic nematodes downregulated several genes involved in proteolysis, response to stimulus, chitin metabolic process, cell surface receptor signal transduction, and lipid metabolic process (Fig. 3c). In addition, *Photorhabdus* infection resulted in substantial increase in the number of upregulated and downregulated genes involved in proteolysis, oxidation-reduction and responses to stress at both 12 and 30 h time-points (Fig. 3d,e). At 12 h, we identified downregulated genes with putative function in Notch signaling, peptidyl amino acid modification, cell recognition, and lymph gland development (Fig. 3d). At 30 h, there was a strong downregulation of genes involved in oxidative phosphorylation, lipid metabolic processes and ribosome function; the latter might be an indication of transcriptional repression (Fig. 3e). We further found upregulation of genes involved in responses to hypoxia and insulin receptor signaling pathway. These data show that infection of the fly with *Heterorhabditis* nematodes and their *Photorhabdus* bacteria causes significant changes in the expression of a large number of protein-coding genes that are involved in key biological processes in the fly. Some of the differentially expressed genes might be important in the regulation of immune function against the pathogens.

### The transcriptome of flies infected by symbiotic *Heterorhabditis* is a combination of the transcriptomes from flies infected by axenic nematodes and *Photorhabdus*

To contrast the transcriptional profiles induced by the nematodes and their bacteria at 12 h and 30 h post-infection, we performed a quadrant plot analysis to identify the genes that are differentially expressed at those two time-points (Additional file 1: Figure S4). We generated three clusters containing differentially regulated genes at both time-points compared to uninfected treatments, genes regulated at 12 h only, and those regulated at 30 h only. Infection with axenic nematodes for 12 h strongly downregulated the genes *CG34424* (5-formyltetrahydrofolate cyclo-ligase), *CG13071* (unknown), *CG33264* (*Or69a*), *CG31748* (*Gr36c*), whereas infection with symbiotic nematodes mostly downregulated genes with unknown function such as *CG43184*, *CG7327*, *CG8960* and *CG42755*. Similarly, *Photorhabdus* infection caused downregulation of several unknown genes, such as *CG13427*, *CG42367* (insect cuticular protein) and *CG13711*. A complete list of the 25 most strongly downregulated genes upon infection with the two pathogens is shown in Additional file 1: Figure S5. We further identified the 25 most strongly upregulated genes in flies infected by axenic nematodes, symbiotic nematodes or the bacteria only (Additional file 3: Dataset S2).

**Fig. 4:**
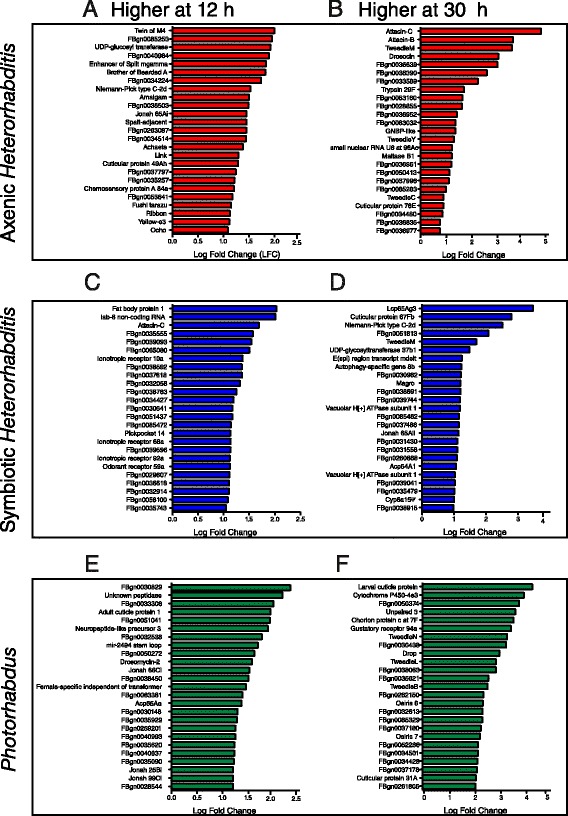
*Heterorhabditis* and *Photorhabdus* induce the expression of diverse subsets of genes in *Drosophila* adults. The 25 most strongly induced genes upon infection with **a**, **b** *Heterorhabditis* axenic nematodes, **c**, **d** *Heterorhabditis* symbiotic nematodes and **e**, **f** *Photorhabdus* bacteria at 12 h and 30 h post-infection. X-axis represents the relative Log-Fold Change (LFC) for each gene after normalization against uninfected controls. All genes have a fold-change higher than 2 (LFC = 0.58 corresponds to 2-fold-change difference)

At 12 h post-infection with axenic worms, we detected increased expression of several genes in the Notch signaling pathway (Fig. 4a). Among this group of genes, the negative regulator *Twin of M4* or *Barbu* showed the highest level of expression followed by a putative CCAT-binding transcription factor, the gene *Enhancer of split mgamma*, a basic Helix-Loop-Helix transcription factor related to *My*c, *Brother of Bearded A*, which has been previously implicated in the fly immune response against bacterial infection [34], *Amalgam*, which codes for an Ig-like C2-type domain-containing protein involved in antigen binding and cell adhesion [35], and the putative enzyme *CG31002* that possesses glucuronosyltransferase activity. Interestingly, we found no increased expression of antimicrobial peptide (AMP) genes (Fig. 4a); only *Attacin C* and *Drosomycin 2* were upregulated upon infection with symbiotic nematodes (Fig. 4c) and *Photorhabdus* bacteria (Fig. 4e), respectively. At 30 h post-infection with axenic nematodes, we found increased expression of the AMP genes *Attacin* and *Drosocin*, a Gram-Negative Binding Protein (*GNBP*)-like gene and several cuticle-related genes such as *Tweedle*, *Cuticular protein 78E* and two genes with chitin-binding domains (*CG7017* and *CG6933*) (Fig. 4b). We also found several highly expressed enzymes including a trypsin-like cysteine protease, a putative AMP-dependent synthetase (*CG4830*), and a putative lipase (*CG5665*).

The transcriptome of flies infected by symbiotic nematodes was a combination of the transcriptomes obtained from flies infected by axenic worms and the bacteria alone. At 12 h post-infection, the gene *Fat body protein 1* was expressed at high levels, followed by the non-coding RNA *Iab-8* that was previously shown to be involved in the regulation of developmental processes [36] (Fig. 4c). We also observed upregulation of several genes coding for ionotropic receptors and the odorant receptor 59a. At 30 h post-infection with symbiotic worms we found increased expression of genes enconding structural components of the cuticle (chitin), such as *Lcp65Ag3*, the *Cuticular protein 67Fb* and the adult cuticular protein *Accessory gland protein 54A1* (Fig. 4d). The gene *Niemann-Pick type C-2d* was also expressed at high levels. Interestingly, this gene codes for a sterol binding protein with Immunoglobulin E-set and MD-2-related lipid recognition domains with a potential function in immune recognition and defense. In addition, there were signatures of transcriptional regulation, as evidenced by the increased expression of the transcription factor *E(spl) region transcript mdelta* and the uncharacterized putative zinc-finger transcription factor *CG14983*. We further detected the expression of *CG16704*, which encodes a putative protein with Proteinase I2 and Kunitz protease inhibitor domains that could be involved in coagulation response or other proteolytic cascades, several subunits for H+ ATPase pumps, a putative transporter (*CG14605*), autophagy-specific gene *Atg8b*, *Tweedle M*, a putative member of the small GTPase family (*CG17819*) and *Jonah 65Aii* protease.

*Photorhabdus* induced a distinct transcriptional profile in the fly compared to the nematodes. At 12 h post-infection we identified a large number of genes with unknown function or identifiable protein domains (Fig. 4e). For example, the gene *CG12998* with unknown function was expressed at the highest level, followed by *CG18179* that codes for a putative peptidase. Other genes that were upregulated at the early phase of infection with *Photorhabdus* included genes coding for cuticular proteins (*Adult cuticle protein 1* and *Adult cuticle protein 65Aa*) and Jonah proteases. We also found a CD36 antigen domain-containing gene (*CG2736*) and the microRNA (miRNA) *Mir-2494* stem loop. At 30 h post-infection with the bacteria, there was increased expression of detoxification genes (*Cytochrome P450-4e3*), the cytokine *Unpaired-3* (component of the Janus kinase/signal transducers and activators of transcription or JAK/STAT pathway) [37], various *Tweedle* genes and two *Osiris* genes (Fig. 4e). These results indicate that infection with *Heterorhabditis* nematodes and *Photorhabdus* bacteria differentially regulates several *Drosophila* genes, many of which have an uncharacterized function in the fly.

### *Heterorhabditis* nematodes and *Photorhabdus* bacteria differentially regulate signature genes in *Drosophila* flies

**Fig. 5:**
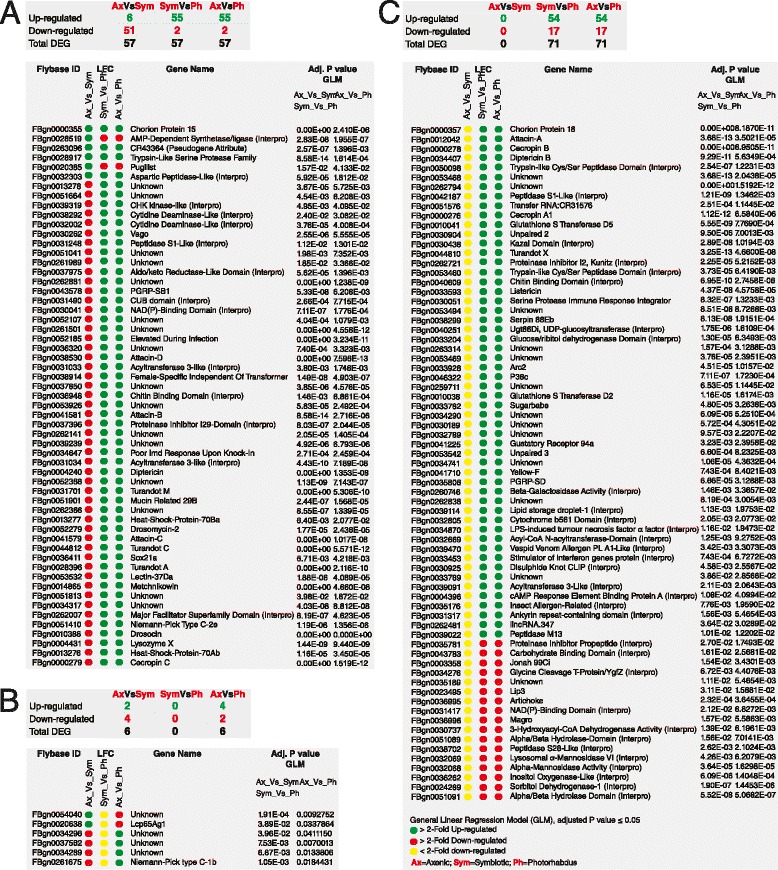
Differential gene expression analysis using DESeq and GLM analysis to compare the different infection types. **a** Filtered list of genes that are common between all three infection types (p < 0.05); **b** Filtered list of genes that are common between the Axenic *Heterorhabditis* vs. Symbiotic *Heterorhabditis* and Axenic *Heterorhabditis* vs. *Photorhabdus* comparisons; **c** Filtered list of genes that are common between the Symbiotic *Heterorhabditis* vs. *Photorhabdus* and Axenic *Heterorhabditis* vs. *Photorhabdus* comparisons. The figure contains *Drosophila* genes with significantly altered expression upon infection of adult flies with the nematodes and their associated bacteria (separately or together), and their corresponding adjusted p-values for the two models used: one to determine the common genes between *Heterorhabditis* (Axenic/Symbiotic) infections and *Photorhabdus* infections, and a second to determine the common genes between Axenic *Heterorhabditis* infection and *Photorhabdus* infection after adjusting for the two time-points. Selected genes included those that appeared in all three comparisons. Red: LFC ≥ 2-fold downregulation; Yellow: LFC ≤ 2-fold downregulation and Green: LFC ≥ 2-fold upregulation

We used the 12 h and 30 h time-points as replicates to perform General Linear Model (GLM) analysis of genes that are differentially expressed upon infection with *Heterorhabditis* nematodes and their mutualistic *Photorhabdus* bacteria, separately or together (Fig. 5 and Additional file 4: Dataset S3). For this, we first compared normalized genes (against uninfected controls) that were differentially expressed upon infection with the pathogens. We then built models to identify the group of genes whose expression levels were significantly affected after comparing the different infection treatments (Axenic *Heterorhabditis* vs. Symbiotic *Heterorhabditis*, Symbiotic *Heterorhabditis* vs. *Photorhabdus*, and Axenic *Heterorhabditis* vs. *Photorhabdus*). Therefore we exclusively selected those genes that showed a significant (2-fold) change in expression (upregulation or downregulation).

We first compared the expression of genes that were affected when comparing all three types of infections using the two time-points as replicates (Fig. 5a). We found that most genes associated with *Heterorhabditis* nematodes (Axenic vs. Symbiotic) were significantly downregulated whereas most genes associated with *Photorhabdus* (Symbiotic *Heterorhabditis* vs. *Photorhabdus* and Axenic *Heterorhabditis* vs. *Photorhabdus*) were significantly upregulated. We observed that several genes encoding AMP (*Attacin*-B, *Attacin*-D, *Attacin*-C, *Cecropin*-C, *Diptericin*, *Drosocin*, *Drosomycin*-2, *Metchnikowin*) as well as *Lysozyme X* were downregulated in nematode-infected flies and upregulated by the bacteria. Other genes including the stress-related genes *Turandot* (*Tot*), *elevated during infection* (*edin*), the Peptidoglycan Recognition Protein gene *PGRP-SB1*, a galactose-specific C-type lectin (*Lectin-37 Da*), and the DNA binding transcription factor *Sox21a*, which have been shown previously to participate in the *Drosophila* immune response [38–43], were also strongly upregulated by *Photorhabdus* but not by *Heterorhabditis* infection. Interestingly, two genes that are involved in metabolic processes, an *AMP-dependent synthetase/ligase* and the putative ATP-binding *Pugilist*, were upregulated in response to *Heterorhabditis* infection and downregulated upon *Photorhabdus* infection.

We then compared the expression of filtered genes that were differentially regulated when comparing Axenic vs. Symbiotic nematode infections and Axenic *Heterorhabditis* vs. *Photorhabdus* infections (Fig. 5b). We observed contrasting gene expression levels between the two comparisons. We also found that although most of the highly regulated genes have an unknown function, gene *Lcp65Ag1* that is involved in the structure of chitin-based cuticle was predominantly upregulated in flies infected by *Heterorhabditis* nematodes [44], whereas gene *Niemann-Pick type C-1b* that is involved in central nervous system development was highly upregulated by *Photorhabdus* infection [45].

Finally, we contrasted the expression of those genes that were significantly affected when comparing Symbiotic *Heterorhabditis* vs. *Photorhabdus* infections and Axenic *Heterorhabditis* vs. *Photorhabdus* infections (Fig. 5c). These comparisons provide insights into the expression of the *Drosophila* genes that are mainly upregulated upon *Photorhabdus* infection, since downregulation of genes in the comparison Axenic vs. Symbiotic *Heterorhabditis* was less than 2-fold. Again we found that AMP genes (*Attacin-A*, *Cecropin A1*, *Cecropin B*, *Diptericin B* and *Listericin*) were upregulated more than 2-fold by *Photorhabdus* infection. We also found that the JAK/STAT pathway cytokines *Unpaired*-2 and *Unpaired*-3 were also significantly upregulated by the bacteria as well as the secreted recognition protein *PGRP-SD*. Other upregulated genes included *Serine Protease Immune Response Integrator* that is involved in the response against bacterial infections [46], the putative regulators of proteolysis *Serpin 88Eb* and *Peptidase M13*, the MAP kinase *P38c* that has been shown to function in the intestine to regulate lipid metabolism and immune homeostasis [47], *Sugarbabe* that encodes a zinc finger protein responsible for the regulation of insulin gene expression in the neurosecretory cells [48], *Glutathione S Transferase D2* that regulates detoxification [49], *Yellow-F* that encodes an enzyme responsible for catalyzing the conversion of dopachrome into 5,6-dihydroxyindole in the melanization pathway [50], and *Gustatory Receptor 94a* that is a candidate taste receptor in *Drosophila* [51]. Downregulated genes by *Photorhabdus* included *Jonah 99 Ci* with putative endopeptidase activity, *Lip3* with putative lipase activity, *Artichoke* that encodes a leucine-rich repeat extracellular matrix protein required for normal morphogenesis and function of ciliated sensilla in *Drosophila* [52], and *Magro* that encodes a lipase A homolog that is secreted from the anterior gut into the intestinal lumen to digest dietary triacylglycerol and hydrolyze cholesterol esters [53].

### Infection with *Heterorhabditis* and *Photorhabdus* alters the transcription of key immune genes in the fly

**Fig. 6:**
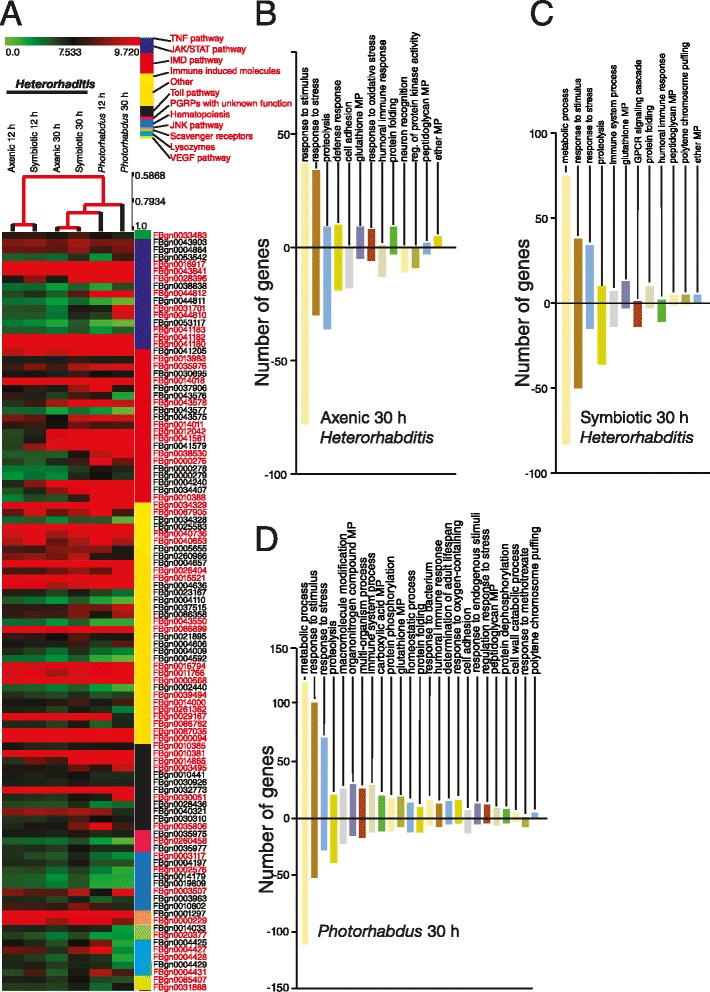
Expression of immune-related genes in *Drosophila* flies infected by *Heterorhabditis* nematodes or their *Photorhabdus* bacteria. **a** Heat map showing immune-related genes that are differentially expressed by *Heterorhabditis* axenic nematodes, symbiotic nematodes and *Photorhabdus* bacteria at 12 h and 30 h post-infection. Genes selected from the Gene Ontology (GO) analysis correspond to the immune response category and have a positive expression level as an indication of their upregulation upon infection with the pathogens. Selected genes were assigned to the following immune pathways or immune-related groups: TNF, JAK/STAT, TOLL, JNK, IMD and VEGF pathways; immune induced molecules, PGRPs with unknown function, hematopoiesis, scavenger receptors, lysozymes and others. GO immune response categories identified in flies infected by **b** *Heterorhabditis* axenic nematodes, **c** *Heterorhabditis* symbiotic nematodes, or **d** *Photorhabdus* bacteria at 30 h post-infection. The Y-axis corresponds to the number of genes for each GO category and their relative level of expression (upregulation or down-regulation). All genes have a fold-change higher than 2 (Log Fold-Change = 0.58 corresponds to 2-fold-change difference)

To identify which immune-related pathways in the fly are regulated upon infection with *Heterorhabditis* and *Photorhabdus*, we generated a heat map to illustrate differences in gene expression levels across all types of infections and time-points (Fig. 6a). We included members of known immune signaling pathways [Tumor Necrosis Factor (TNF), JAK/STAT, Immune Deficiency (IMD), Toll, Jun-N-terminal Kinase (JNK), and Vascular Endothelial Growth Factor (VEGF)], genes involved in hematopoiesis, putative immune recognition receptors, scavenger receptors, lysozymes and several genes with putative immune function.

In the IMD pathway [54], the PGRP genes *PGRP-LB* and *PGRP-LC* were strongly induced by *Photorhabdus* and symbiotic *Heterorhabditis*, but not by axenic nematodes. *PGRP-SC1a* was upregulated by symbiotic worms and downregulated by axenic worms and the bacteria alone, whereas *PGRP-SB2* was downregulated by all three pathogens. The transcription factor *Relish* was strongly induced by both nematodes and bacteria. AMP genes regulated by the IMD pathway were induced at different levels by the pathogens. For example, *Cecropin A1/A2* levels decreased at 30 h by *Photorhabdus* and axenic nematodes, but not by symbiotic nematodes. *Attacin C* and *Drosocin* were upregulated at high levels by *Photorhabdus* infection and at low levels by axenic and symbiotic worms. Similarly, several members of the Immune-induced molecules (IIM) family were differentially regulated by infection with the nematodes and their bacteria. Genes *IIM1-4* were strongly upregulated upon infection with all three pathogens, while *IIM2 and 3* expression decreased at 30 h post-infection with *Photorhabdus*.

In the Toll pathway [55], we found differential expression of several known pattern recognition receptors. These included *GNBP-3* that was highly expressed by axenic and symbiotic nematodes at both time-points and by *Photorhabdus* at 12 h post-infection, *PGRP-SA* and *PGRP-SD* that were upregulated by symbiotic nematodes at 30 h and *Photorhabdus* at 12 h, and *PGRP-LD* that was downregulated by all pathogens at both time-points. We also found that the cytokine *Spaetzle* was expressed by infection with symbiotic nematodes and *Photorhabdus* at 30 h post-infection and the Toll immune-regulated protein Fondue was expressed by all types of infection, whereas *Metchnikowin* was upregulated by infection with *Photorhabdus* at both time points.

In the JAK/STAT pathway [56], the receptor *Domeless*, the *STAT* transcription factor and the effector gene *Virus-induced RNA1* (*Vir-1*) were strongly induced by both types of *Heterorhabditis* nematodes and their *Photorhabdus* bacteria at both time-points. *Tot* genes and genes encoding thioester-containing proteins (TEPs), which participate in opsonization of microbes [57], were differentially expressed by the pathogens. These data suggest that *Heterorhabditis* and *Photorhabdus* may induce different stress responses and cellular immune reactions during infection of *Drosophila* adult flies.

In the JNK pathway [58], which controls genes that participate in wound healing and cellular immune processes including hemocyte proliferation and differentiation [59], we found that both axenic and symbiotic *Heterorhabditis* as well as *Photorhabdus* infection upregulated *Kayak* (the *Drosophila Fos* homolog), while infection with symbiotic nematodes and the bacteria alone upregulated *Basket* (the *Drosophila* JNK homolog) at 12 and 30 h post-infection, respectively.

Among the genes involved in hematopoiesis, we found that *Serpent* was upregulated by infection with axenic nematodes at 12 h and by infection with symbiotic nematodes and *Photorhabdus* at 30 h post-infection. Among the genes in other categories, we found several genes involved in melanization, such as *Serine protease-7*, which is upregulated by infection with symbiotic nematodes and *Photorhabdus*, while *Gram-positive specific serine protease* and *Black cells* were downregulated by infection with symbiotic nematodes and their bacteria. We further found that transcription of lysozyme-coding genes was also altered after infection with the pathogens.

### *Heterorhabditis* and *Photorhabdus* affect several immune processes in the fly

We then determined immune system functions derived from the immune response GO categories in flies infected for 30 h by axenic *Heterorhabditis*, symbiotic *Heterorhabditis* or *Photorhabdus* bacteria alone. We found that infection with axenic nematodes upregulated 37 genes and downregulated 78 genes in the GO category “response to stimulus” (Fig. 6b). Upregulated genes included several *Heat shock protein* genes (*Hsp*22, *Hsp*23, *Hsp68*, *Hsp70*, *Hsp73, Hsp74*), *PGRP-SD*, and *Nuclear Factor-kappa-B* (*NF-κB*) *p110*. Downregulated genes included *Lectin-46Ca*, *PGRP-LE*, *PGRP-SC1a/b*, *PGRP-SC2*, *Toll 7*, *TEP I*, and several lysozyme genes. The second most affected category included genes involved in “stress response”. Here we found 34 upregulated and 30 downregulated genes. We also observed that infection with axenic nematodes affected the expression of genes involved in oxidative stress (*Peroxiredoxin*, *Peroxidasin*, *Glutathione peroxidase*, *Stress-activated protein kinase JNK*, *Hsp22* and several *Glutathione S-transferases*). Strikingly, axenic nematode infection downregulated all genes in the cell adhesion category. Some of those included *Down Syndrome Cell Adhesion Molecule (DSCAM) 3* isoform, *Epidermal growth factor receptor*, *Pointed* and *Fasciclin 2*. Genes that regulate kinase activity including the stress-activated protein kinase JNK and several members of the *Stellate* gene family were also downregulated.

Infection with symbiotic *Heterorhabditis* also regulated *Drosophila* genes involved in response to stimulus (Fig. 6c). We found 38 upregulated and 50 downregulated genes. Upregulated genes included genes coding for Hsp and several PGRPs (*PGRP-SD*, *PGRP-SA*, *PGRP-LF*, *PGRP-LB*), the NF-κB subunit *p110*, a metallopeptidase (*CG11865*) with predicted metalloendopeptidase activity and genes with disulphide-knot and trypsin-like cysteine/serine peptidase domains (*CG9733* and *CR30374*). Downregulated genes included *PGRP-SB2*, *TepI*, several *lysozyme* genes, and several genes involved in GPCR signaling (neurotransmitter receptors, *Methuselah* receptors and diuretic hormone receptors). Interestingly, we found that most genes involved in proteolysis were downregulated by the symbiotic worms as well as genes coding for proteases (e.g. FBgn005236, *CG30288*, *CG33459*), and in particular those that share common endopeptidase activity domains.

A large number of fly genes was affected by *Photorhabdus* infection (Fig. 6d). In the “response to stimulus” category, 102 genes were upregulated and 51 were downregulated. Upregulated genes included several PGRPs (*PGRP-SB1*, *PGRP-SA*, *PGRP-LF*, *PGRP-LC*, *PGRP-LB*, *PGRP-SC2*, *PGRP-LA*) and several genes involved in the activation and signaling of immune pathways such as *NF-κB* subunit p110, *Puckered*, *Pelle*, *Kayak*, *Toll4*, *TNF-receptor associated factor 4*, *Domeless*, *Hopscotch*, *MAP kinase-activated protein kinase 2*, and *Mitogen-activated protein kinase ERK*. In addition, *TEPI, and TEPIV*, *Serine protease 7*, *Multidrug-Resistance like protein 1* and several *Methuselah* genes were also upregulated. Genes downregulated by *Photorhabdus* infection included several receptors (*PGRP-LD*, *PGRP-SC1a/b*, *Fibroblast-growth factor receptor homolog 2*, *Methuselah-9* and *GNBP-3*) as well as several lysozyme genes.

At 12 h post-infection, we found that infection with axenic and symbiotic *Heterorhabditis* differentially regulated genes involved in metabolic and proteolytic processes; however their number was substantially reduced compared to those regulated by nematode infection at 30 h post-infection. Also, infection with symbiotic worms upregulated several genes involved in oxidative stress and regulation of hypoxia. In general, we found a higher ratio of upregulated/downregulated immune genes in flies infected by symbiotic worms at 12 h compared to the 30 h time-point. In sharp contrast, there was a lower ratio of upregulated/downregulated genes involved in metabolism and response to stress in flies infected by *Photorhabdus* as well as downregulation of GPCR signaling and adhesion genes (Additional file 1: Figure S6).

## Discussion

*Heterorhabditis* nematodes and their mutualistic *Photorhabdus* bacteria, are excellent tools to probe the genetic and molecular basis of anti-nematode and antibacterial immunity in insects [7]. Here we have shown that several types of genes in *Drosophila* are differentially regulated upon infection with these pathogens. Our goal was to identify the number and nature of genes that are upregulated or downregulated in adult flies by *Heterorhabditis* and *Photorhabdus* infection. We have found that infection by the two pathogens regulates different sets of genes and the signaling pathways they control in the fly. This implies that the nematodes and their associated bacteria may employ distinct strategies to interfere with innate immune defense mechanisms in the fly.

A recent genome-wide transcriptional analysis of *Drosophila* larvae infected by *Heterorhabditis* symbiotic nematodes identified significant upregulation of several immune genes as well as genes with putative immune function, while genes with lower induction fell into three specific pathways: the oocyte maturation pathway, the Wnt signaling pathway and the ubiquitin-mediated pathway [18]. Another transcriptomic analysis of *Drosophila neotestacea* adult flies infected by the nematode parasite *Howardula aoronymphium* reported upregulation of several genes with putative immune function (lectins, fibrinogen-like domain-containing proteins, clotting activity, chitin metabolism), but failed to detect significant upregulation of specific immune-related genes [60]. Here we find that infection of *D. melanogaster* adult flies with axenic or symbiotic *Heterorhabditis* nematodes, or *Photorhabdus* bacteria alone generates distinct transcriptional profiles. RNA-Seq transcriptional analysis of the fly response to *Heterorhabditis* suggests that nematode infection leads to major changes in the transcription of a large number of *Drosophila* genes, several of which are involved in vital physiological functions in the fly. Although the exact pathological effects of *Heterorhabditis* and *Photorhabdus* on *Drosophila* adult flies are currently unknown, we would expect that migration/replication of the pathogens within the fly as well as secretion of virulence factors or secondary metabolites would result in a more general metabolic stress response to the pathogens.

Increased stress response to *Heterorhabditis* and *Photorhabdus* is also evident by the upregulation of Hsp genes upon infection with the pathogens. Upregulation of Hsp genes could potentially serve as part of a core stress response program in the fly against nematode-bacteria complexes. Here we also find that the induction of Tot genes, which are mainly induced under stress conditions and participate in *Drosophila* stress tolerance [38, 39], differs among the different types of infection. This indicates that *Heterorhabditis* and *Photorhabdus* might induce different stress responses during infection of the fly. The regulation of proline and glutathione metabolism pathways in the fly could indicate a potential role in protection against increased cellular stress or mechanical injury (e.g. tissue disruption, cell death) in response to infection with the nematodes and their bacteria [61, 62]. The identification of genes coding cysteine proteases, synthetases and lipases that are associated with lipid metabolism also suggests that these enzymes might be involved in the response of the fly to internal tissue damage or form a defense mechanism against the pathogens [63].

*Photorhabdus* bacteria are members of the Enterobacteriaceae family, and as such are closely related to medically important pathogens including *Escherichia coli*, *Salmonella* and *Yersinia* spp., potentially sharing common mechanisms [64]. Although transcriptomic analyses of the *Drosophila* response to *Salmonella* or *Yersinia* pathogens have not been performed thus far, previous transcriptomic studies have reported that systemic infection with *E. coli* bacteria resulted in the activation of target genes in Toll and Imd pathways [65, 66]. In particular, the authors found several *Relish*-dependent and *Spaetzle*-dependent genes that were significantly upregulated upon infection with *E. coli*, as well as genes coding for AMP, putative microbial pattern-recognition proteins, proteases and their inhibitors [65]. Other genes were assigned to functions related to phagocytosis, melanization and coagulation, wound healing, produciton of reactive oxygen species and ion sequestration [66]. In another study, injection with a pathogenic strain of *Pseudomonas aeruginosa* led to significant transcriptional upregulation of genes related to stress, hemocyte-proliferation, putative catabolism genes and genes predicted to regulate proton transport; whereas downregulated genes included serine proteases, tissue morphogenesis and olfactory genes, GPCR and some humoral immunity genes [67, 68].

Here we found that flies infected by *Photorhabdus* generated the highest number of sequence reads. This could reflect the ability of these pathogens to interfere with gene transcription in the fly. This assumption is supported by our findings showing that infection with *Photorhabdus* downregulates most *Drosophila* genes at early times post infection. Given that the bacteria are released by their nematode vectors within a few hours after the worms gain access to the insect [13], our results indicate that a potential strategy of *Photorhabdus* to overcome the insect immune response could be the induction of an early strong transcriptional downregulation of key genes in the fly. Current results also indicate that *Photorhabdus* can affect gene transcription in *Drosophila* at late times of infection when flies have started to succumb to the bacteria. In particular, a signature pattern unique to *Photorhabdus*-infected flies was the enrichment of 60 downregulated genes involved in ribosomal function and structure. This could be an indication of translational repression during the late phase of infection that could form a *Photorhabdus* tactic to enhance immunosuppression in the fly [49]. We further found that infection with *Heterorhabditis* and *Photorhabdus*, separately or together, downregulates several genes that participate in folate biosynthesis. Given that folate plays a crucial role in DNA and protein synthesis and cell-mediated/humoral immune responses are especially affected by folate deficiency [69, 70], this could probably imply that the nematodes and their bacteria have the ability to interfere with DNA synthesis and nucleotide biogenesis in the fly. Should this be the case, the pathogens could utilize the host-cell machinery to their advantage in facilitating their survival, spread and replication in the fly. Also, in agreement with previous findings that *Photorhabdus* bacteria interact with the insect gut [71, 72], here we found that infection of flies with *Photorhabdus* differentially regulates several genes that are mainly expressed in the *Drosophila* gut and participate in immune homeostasis in this tissue [73–75].

Our RNA-Seq analysis detects upregulation of a large number of AMP genes in flies infected by *Photorhabdus*. AMP are important molecules in host defense [76]. In *Drosophila* most AMP appear in the hemolymph within a few hours after microbial challenge, their concentrations increase rapidly and some persist for several hours [77]. These results suggest that AMP may play a role in the immune response of *Drosophila* against *Photorhabdus* by slowing down bacterial replication and spread in the fly; however, AMP upregulation proves ultimately an ineffective defense against those pathogens since flies succumb to *Photorhabdus* infection within a few days. We have previously reported that infection with a low number of *Photorhabdus* cells fails to upregulate AMP expression in infected flies [17]. Given that in the current study we have injected flies with 5-7 times more bacterial cells compared to the previous study, we suspect that *Photorhabdus* induces AMP gene transcription in a dose-dependent manner. This will be a subject for future investigations. In contrast to our current findings, a previous study has reported that infection with the pathogenic bacteria *P. aeruginosa* significantly downregulates the transcription of AMP genes in *Drosophila* adult flies during the initial stages of infection, which probably forms a mechanism that facilitates replication of the pathogen in the insect hemolymph, which is a hostile environment for bacterial growth, and promotes bacterial survival and pathogenesis in the host [67, 68].

Information on molecules with anti-nematode activity in insects is currently lacking [7]. The use of the *Drosophila-Heterorhabditis* model together with RNA-Seq can provide important clues for potential molecules that might act against nematode infection in insects. TEPs are well-conserved proteins that participate in the immune response of animals against bacteria and parasitic protozoans [57, 78]. Recent studies in *Drosophila* showed that TEPs are not involved in the defense against certain bacterial and fungal pathogens [79]. It was hypothesized that TEPs are likely to participate in the immune function against pathogens attacking the fly through the cuticle, such as nematode parasites. In addition, a recent transcriptomic study on *Drosophila* larvae reported that certain TEPs were differentially regulated upon infection with symbiotic worms [18]. Our RNA-Seq data show that TepI was downregulated whereas TepII and TepIV were strongly upregulated at early and late times after infection with the nematodes and their bacteria. These findings suggest that different TEP molecules may participate in the immune function of *Drosophila* larvae and adult flies against nematode infection. Future research will focus on the functional characterization of TEP anti-nematode and antibacterial properties in *Drosophila* in response to *Heterorhabditis* and *Photorhabdus* as well as to other nematode-bacteria complexes.

Recent progress in understanding the molecular basis of organismal responses to hypoxia has led to the identification of hypoxia-inducible transcription factors (HIF) and their hydroxylation by the prolyl hydroxylase enzymes [80]. The prolyl hydroxylation process is central to the regulation of hypoxia-induced genes during inflammation. In addition, recent findings have emphasized the regulatory role for HIF in the hypometabolism of insects [81]. Here we found significant downregulation of genes encoding prolyl hydroxylase enzymes in flies infected by *Heterorhabditis* axenic nematodes. This indicates that flies might be able to diminish metabolic functions or alter their metabolic state through the prolyl hydroxylation mechanism in order to withstand nematode attacks by diverting energy resources to enhance their immune response against the parasites. Similarly, because glycosylation is a key process in the generation of extracellular matrix components and mucins [82], upregulation of glycosyltransferase enzymes upon infection of the fly with axenic *Heterorhabditis* could function as a mechanism to repair tissue damage that is likely caused by nematode infection.

Infection of adult flies with axenic or symbiotic *Heterorhabditis* upregulates genes coding for glutamate ionotropic receptors and putative sodium channels, which have previously been reported to participate in nociception [83–85]. This indicates potential changes in this neural function of the fly during nematode infection, since ionotropic receptors are involved in sensing putative external and internal cues [86]. It was recently reported that class IV neurons are required for a nociceptive behavioral response of *Drosophila* larvae against infections by parasitoid wasps, and such neural reactions are mostly elicited by attacks during which the cuticle is penetrated by the wasp [87]. Therefore, it is possible that *Drosophila* employs similar neural mechanisms to process noxious stimuli in response to tissue damage or other potent chemical/mechanical stimulation caused by nematode invasion or migration in the fly. This could represent a neuronal function of *Drosophila* to sense the presence of certain chemical cues or metabolites produced by the nematodes [64]. It has been shown previously that entomopathogenic nematodes secrete peptidases and peptidase inhibitors that may target and degrade insect tissues or actively suppress important host immune defenses, such as prophenoloxidase activation and melanization [7, 88, 89]. Interestingly, the *H. bacteriophora* genome encodes 19 putative peptidase and 9 peptidase inhibitors [90]. In mammals, it has been shown that biogenic amines that are produced during pathological conditions can be detected by trace amine-associated receptors, a class of GPCR, present in erythrocytes [91].

Although recognition molecules that detect bacterial, fungal and viral pathogens have been identified in *Drosophila* [92], it is currently unknown whether and how the fly immune system detects the presence of nematode parasites, and what specific molecules could be responsible for this function. Here we have found that infection with *Heterorhabditis* nematodes significantly upregulates genes (e.g. *Acp54A1*, *Cpr78E*, *Cpr67Fb*, *Lcp65Ag1*, *Lcp65Ag3* and *Tweedle* family genes) coding for structural components of chitin-based cuticle [44, 93]. Notably, *Cpr78E* and *Tweedle* family genes encode proteins that are secreted by ectodermal tissues including epidermis, foregut and tracheae and previously they were shown to contribute to cuticle formation. Sequence analysis predicts that they possess chitin-binding activity, which renders these proteins as potential candidate recognition molecules for detecting nematode invasion in the fly or migration of the parasites within fly tissues [94–96]. Interestingly, oral infection of *D. melanogaster* larvae with *Erwinia carotovora carotovora 15* bacteria resulted in the regulation of several genes encoding chitin-binding proteins and in particular *Tweedle* genes in the tracheae. This was attributed to the interaction between the bacteria and the chitinous layer that protects the tracheae [97].

## Conclusions

Taken together, transcriptome profiling through RNA-Seq provides an excellent approach for the precise assessment of transcript levels and transcript isoforms in the *Drosophila* model of infection and immunity. RNA-Seq analysis of *Drosophila* adult flies infected by *Heterorhabditis* nematodes and their mutualistic *Photorhabdus* bacteria reveals transcriptional changes in the regulation of a large number of genes, many of which have not been shown previously to participate in immune processes against pathogenic infections. Many of those genes that are differentially regulated upon *Heterorhabditis* or *Photorhabdus* infection are predicted to be involved in metabolic functions, stress responses, DNA/protein synthesis and neuronal activities. In addition, we have identified *Drosophila* genes with potential role in nematode detection and molecules with potential anti-nematode properties. Many of those molecules provide an excellent platform of candidate factors for the functional characterization of the *Drosophila* immune response against nematode-bacterial complexes. Future studies using the *Drosophila*-*Heterorhabditis*-*Photorhabdus* model promise to reveal not only how pathogens evolve virulence but also how two pathogens (nematode and bacteria) can synergize to exploit a common host.

## Methods

### Fly stocks

Oregon R adult flies were used for the transcriptomic analyses. The strain was kindly provided by Prof. Jean-Marc Reichhart (UPR9022 of CNRS, Institute of Molecular and Cellular Biology, Strasbourg, France). Flies were reared on instant *Drosophila* diet (Formula 4-24 *Drosophila* medium) supplemented with yeast (Carolina Biological Supply), and maintained at 25 °C and a 12:12-h light:dark photoperiodic cycle. Equal number of male and female adult flies aged 4-6 days old were used in infection assays with the nematodes and their bacteria.

### Nematodes and bacteria

*Heterorhabditis bacteriophora* TT01 strain entomopathogenic nematodes were amplified in fourth instar larvae of the wax moth *Galleria mellonella* using the water trap technique [98]. To confirm lack of *Photorhabdus* bacteria in *Heterorhabditis* nematodes (axenic), the worms were homogenized and the lysate was spread on selective media. Fresh IJ worms were collected and prepared through pelleting, washing and re-suspending in sterile distilled water. IJ nematodes carrying or lacking *Photorhabdus* bacteria were used 1-2 weeks after collection from the water traps. *Heterorhabditis* numbers were estimated by counting the average nematode density present in ten individual 50 μl drops of water using a stereo-microscope.

The Gram-negative insect pathogenic bacteria *Photorhabdus luminescens* subsp. laumondii (strain TT01) were used for fly infections. Bacteria were cultured in Luria-Bertani broth (LB) and incubated for 18-24 h at 30 °C. Bacterial cultures were centrifuged at 4 °C, pelleted, washed in 1x sterile phosphate-buffered saline (PBS) and re-suspended in PBS. Bacterial density was measured using a NanoDrop™ 2000c (Thermo Fisher Scientific) and a 10x serial dilution plating technique.

### Infection assays

Nematode infection assays in *Drosophila* adult flies have been described in detail previously [27]. Briefly, nematode infections were carried out using nested 5 ml cups (Solo®) and filter papers (Whatman) that supported 10-15 adult flies per group. A 500-700 μl solution containing symbiotic or axenic *Heterorhabditis* nematodes was added to each container (100 IJ/fly). Flies treated for 30 h with sterile water devoid of nematodes were used as negative controls. Infected and control flies were kept at 25 °C. A PBS suspension (18.4 nl) containing *Photorhabdus* bacteria was injected into *Drosophila* adult flies at the lateral anterior side of the thorax through nano-injection (Nanoject II - Drummond Scientific). The number of *Photorhabdus* cells delivered into each fly was approximately 500-700 colony-forming units (CFUs). Control samples involved PBS injected flies.

### RNA isolation

RNA was extracted from 40 adult flies infected by *Heterorhabditis* axenic nematodes, symbiotic nematodes, *Photorhabdus* bacteria only as well as from uninfected controls. Samples were collected at 12 and 30 h post infection. Total RNA was extracted using the Prep*Ease* RNA spin kit (USB) following the manufacturer’s instructions. Briefly, flies were homogenized using sterile plastic pestles and RNA was extracted using a silica-based column system including a DNAse treatment step for 15 min. Total RNA was re-suspended in 40 μl of sterile nuclease-free water. RNA concentration was measured using a Nanodrop. RNA integrity and quality were assessed using a Bioanalyser (Agilent Technologies).

### Library preparation and RNA sequencing

Separate libraries for the four experimental conditions (flies infected by *Heterorhabditis* axenic or symbiotic nematodes or *Photorhabdus* bacteria alone as well as uninfected controls) were prepared using the TruSeq RNA sample preparation kit V.2 (Illumina) and rRNA-depleted total RNA as template. Ribosomal RNA present in total RNA samples was removed prior to library construction using the Ribo-Zero™ rRNA Removal Kits (Epicentre Technologies). Briefly, 10 μg of total RNA were obtained through two rounds of rRNA reduction. For the first round, oligos from the Human/Mouse/Rat and Gram-Negative Bacteria Ribo-Zero™ kits were mixed in equal parts. The resulting RNA was then taken through a second round of reduction using the Human/Mouse/Rat Ribo-Zero™ kit. Depleted RNA was then fragmented and reversed transcribed using random hexamers. Single stranded fragments were end-repaired, and phosphorylated for the A-tailing step for index adaptor ligation. Fragments were PCR amplified to create linear fragments containing adaptor sequences (6 nucleotide indexes) to initiate cluster generation and sequencing. The DNA was purified between enzymatic reactions and the size selection of the library was performed using AMPure XT beads (Beckman Coulter Genomics). All seven samples were multiplexed and run on a single lane on a flow cell of an Illumina HiSeq 2000, resulting in seven libraries with ~25 million paired-end reads. RNA sequencing was performed at the Institute for Genome Sciences (University of Maryland School of Medicine).

### Alignment reads and coverage analysis

Seven samples representing the three infection types and a normalization control were collected at two time points and processed for sequencing. One hundred and one base-pair long reads were generated and then introduced into TopHat using the *Drosophila melanogaster* Reference Genome version BDGP5 to map out reads to gene models predicted by the fly genome [99]. Bowtie was then used to assemble transcripts by mapping and identifying splice junctions [100]. For quality control, reads were only allowed to have up to two mismatches per 30 base pairs, and reads that matched to more than 25 locations were removed from the analysis. For the coverage analysis, BAM files generated using TopHat were used to determine the total number of reads, the number of mapped reads and the percentage of mapped reads to the *Drosophila melanogaster* genome, which allowed direct comparisons of samples among the different treatments.

### Differential gene expression analysis

BAM alignments generated by TopHat were then utilized to calculate Reads Per Kilobase of gene per Million mapped reads values (RPKM), and indexed using in-house scripts and tools for each individual gene model and sample. RPKM analysis calculates gene expression differences by normalizing the read counts for the total length of the gene and the number of mapped sequencing reads [101]. These RPKM values were then normalized using the 75^th^ quantile normalization to ensure similar distributions across all samples. The Fold-Change for each individual gene was calculated using the normalized RPKM values. The differentially expressed genes were determined after applying a minimum read count cut-off of 10, Log fold-change cutoff of 0.58 (minimum one fold-change) and RPKM value greater than 0.1. RPKM data were used to create quadrant plots using log fold-changes (LFC) values.

### Transcript analysis using CUFFLINKS

BAM files generated using TopHat were uploaded into the Cufflinks transcriptome identification tool [28], to assemble aligned RNA-Seq reads into predicted transcripts and calculate relative abundances. RNA-Seq fragment counts were used to calculate FPKM values (Fragments Per Kilobase of exon per Million fragments mapped) to estimate transcript abundance.

### Differential transcript analysis using Cuffdiff

BAM files generated by TopHat were uploaded onto Cuffdiff (component of the Cufflinks package) to estimate differential expression between samples (grouped by experimental condition) at the transcript level. The statistical model used assumes that the number of reads produced by each transcript is proportional to its abundances. It also utilizes transcript expression from replicates to estimate variance and calculate the significance of observed changes in expression. The significance of differential expression of transcripts belonging to the same gene across the two conditions (infected vs. uninfected) was tested using the negative binomial (NB) distribution. A cutoff of False Discovery Rate (FDR) less than 0.05, FPKM > 10 and Log Fold-Change of 0.5 was used to select significantly differentially expressed transcripts. The data generated by Cuffdiff were used to calculate the distribution of read counts for each transcript, volcano plots (FDR vs LFC), and F plots (FPKM_sample1_ vs FPKM_sample2_).

### Gene ontology analysis (GO)

GO analysis was performed using the list of differentially expressed genes to search the DAVID web service to assign GO categories to the identified genes. We selected the Molecular Function, Cellular Compartment, Biological Process, Pathway Analysis and Protein Domains categories for our analysis.

### Differential gene expression analysis using DESeq and General Linear Model (GLM)

The alignment files generated from TopHat were used to compute read counts for each gene in the reference annotation (Berkeley Drosophila Genome Project release 5). The read counts were computed for each sample using the HTSeq (v0.5.3) library available for Python. The read counts were then used as input for DESeq (v1.10.1). DESeq is a R bioconductor package which estimates the variance-mean dependence in count data from high-throughput sequencing assays, normalizes the count data for library sizes and dispersion, and tests for differential expression based on a model using the negative binomial distribution. The samples were clustered using the normalized values to identify outliers (if any). A GLM was used wherein the infection type (Symbiotic *Heterorhabditis*, Axenic *Heterorhabditis*, *Photorhabdus* only) and time after infection (12 and 30 h) were treated as the explanatory variables. The p-values were generated using the nbinomGLMtest in DESeq and adjusted using the Benjamin-Hochberg method to control for false discovery. The significant differentially expressed genes were identified for multiple comparisons after applying significance cut-offs (adjusted p-value ≤ 0.05 and absolute (fold-change) ≥ 2, or ≤ 2).

### qRT-PCR validation

To validate differentially expressed genes, we selected seven candidate genes based on significant fold differences across all samples and analyzed their relative mRNA levels using qRT-PCR, as previously described [17]. Five adult flies from each treatment were frozen at 12, and 30 h after infection. Total RNA was extracted using the PrepEase RNA spin kit (USB) following the manufacturer’s instructions. RNA samples were re-suspended in 40 μl of sterile nuclease-free water and RNA concentrations were measured using a Nanodrop (Thermo Scientific). Complementary DNA (cDNA) synthesis was synthesized using the High Capacity cDNA reverse transcription kit (Applied Biosystems). cDNA samples were diluted 1:10 in nuclease-free water and 1 μl was used as template for qRT-PCR experiments using the EXPRESS SYBR® GreenER kit with Premixed ROX (Invitrogen). All experiments were performed on a Mastercycler® ep realplex^2^ (Eppendorf) and twin-tec real-time PCR 96-well plates following the manufacturer’s instructions. Annealing temperatures were optimized using gradient reactions. All primers produced single amplicons as evidenced by dissociation curves (melting curve analysis). Technical duplicates were run for each sample and set of primers, and a total of four biological samples were used for each treatment. The cycling conditions included 50 °C for 2 min, 95 °C for 2 min, 40 cycles of 95 °C for 15 sec and an annealing step for 45 sec. For each sample, the amount of mRNA detected was normalized to mRNA values of the control housekeeping gene *Ribosomal protein L32* (*RpL32*, *CG7939*). Normalized data were used to quantify the relative level of a given mRNA according to cycling threshold analysis (ΔCt), hence data were expressed as the ratio 2^CT(RpL32)^/2CT^(Gene)^. Data are presented as a ratio between infected versus PBS injected flies (negative controls for bacterial infections) or untreated flies (negative controls for nematode infections). The list of primers is given in Additional file 1: Table S1.

### Statistical analysis

qRT-PCR results represent the means and standard deviations of relative values from three biological replicates. Data were statistically analyzed using a one-way analysis of variance (ANOVA) with a Tukey *post-hoc* test for multiple comparisons (GraphPad Prism).

### Data access

The raw sequence data that were generated in the course of this research are made publicly available. Paired-end sequencing data of the *D. melanogaster* transcriptomes have been deposited to the NCBI Gene Expression Omnibus (GEO, http://www.ncbi.nlm.nih.gov/geo/) database and are available under the accession number GSE61466 http://www.ncbi.nlm.nih.gov/geo/query/acc.cgi?token=szetgcuwtvsvhyf&acc=GSE61466.

## Additional files

Additional file 1:
**Text description.** **Figure S1.** Gene coverage density plot. **Figure S2.** Quantitative real-time RT-PCR validation. **Figure S3.** Infection of *Drosophila* flies with *Heterorhabditis* nematodes or their *Photorhabdus* bacteria induces diverse physiological responses. **Figure S4.** Quadrant plots showing expression patterns in adult flies infected by nematodes or their bacteria. **Figure S5.** Infection of *Drosophila* flies with *Heterorhabditis* or their *Photorhabdus* suppresses the expression of several genes. **Figure S6.** Gene Ontology analysis. **Table S1.** List of primers used for quantitative real-time RT-PCR validation. Additional file 2:
**Gene lists corresponding to the KEGG and PANTHER pathway analysis for** ***Drosophila*** **wild-type adult flies infected by** ***Heterorhabditis*** **axenic or symbiotic nematodes or** ***Photorhabdus*** **bacteria at 12 and 30 h time-points.** Gene identity and description corresponding to pathway categories (KEGG), which are shown in Fig. 2 (panels a, b and c) and Additional file 1: Figure S3. Additional file 3:
**Gene lists corresponding to the Quadrant Plot analysis** **(Additional file** **1** **: Figure S4) used to generate Fig.** **4.** The 25 most strongly induced *Drosophila* genes upon infection of wild-type adult flies with *Heterorhabditis* axenic nematodes, *Heterorhabditis* symbiotic nematodes or their mutualistic *Photorhabdus* bacteria at 12 h and 30 h following immune challenge. Additional file 4:
**Complete filtered list of genes obtained from the General Linear Model analysis** **(Fig.** **5)** **using** ***Drosophila*** **reads from Symbiotic** ***Heterorhabditis*** **infections to compare to those from Axenic** ***Heterorhabditis*** **infections and** ***Photorhabdus*** **infections.** Additionally, we used *Drosophila* reads from Axenic *Heterorhabditis* infections to compare to those from *Photorhabdus* infections. The data from the two time points (12 and 30 h) for each infection type were used as biological replicates. We used the infection type as the explanatory variable and each gene is accompanied by its Log2 Fold-Change and p values. The table shows genes that are differentially regulated with an absolute 2-fold (or higher) change upregulation or downregulation.

## Abbreviations

AMP: Antimicrobial peptide
ANOVA: Analysis of variance
ATP: Adenosine triphosphate
cDNA: complementary DNA
CFU: Colony-forming units
DAVID: Database for annotation, visualization and integrated discovery
DSCAM: Down syndrome cell adhesion molecule
FDR: False discovery rate
FPKM: Fragments Per Kilobase per Million
GEO: Gene expression omnibus
GNBP: Gram-negative binding protein
GLM: General linear model
GO: Gene ontology
GPCR: G-protein coupled receptor
GTP: Guanosine triphosphate
HIF: Hypoxia-inducible transcription factors
Hsp: Heat shock protein
IIM: Immune-induced molecules
IJ: Infective Juvenile
IMD: Immune deficiency
JAK/STAT: Janus kinase/signal transducers and activators of transcription
JNK: Jun-N-terminal kinase
KEGG: Kyoto encyclopedia of genes and genomes
LFC: Log fold-changes
miRNA: microRNA
NB: Negative binomial
NF-κB: Nuclear Factor-kappa-B
PANTHER: Protein analysis through evolutionary relationships
PBS: Phosphate-buffered saline
PGRP: Peptidoglycan recognition protein
qRT-PCR: Quantitative real-time RT-PCR
RNA-Seq: RNA Sequencing
RPKM: Reads Per Kilobase of gene per Million mapped reads values
rRNA: Ribosomal RNA
TEP: Thioester-containing Proteins
TNF: Tumor necrosis factor
Tot: Turandot
VEGF: Vascular endothelial growth factor
Vir-1: Virus-induced RNA1

## References

[1] Medzhitov R (2007). Recognition of microorganisms and activation of the immune response. Nature.

[2] Glavis-Bloom J, Muhammed M, Mylonakis E (2012). Of model hosts and man: using *Caenorhabditis elegans, Drosophila melanogaster* and *Galleria mellonella* as model hosts for infectious disease research. Adv Exp Med Biol.

[3] Brivio MF, Mastore M, Pagani M (2005). Parasite-host relationship: a lesson from a professional killer. Invertebr Surv J.

[4] Dionne MS, Schneider DS (2008). Models of infectious diseases in the fruit fly *Drosophila melanogaster*. Dis Model Mech.

[5] Limmer S, Quintin J, Hetru C, Ferrandon D (2011). Virulence on the fly: *Drosophila melanogaster* as a model genetic organism to decipher host-pathogen interactions. Curr Drug Targets.

[6] Rämet M (2012). The fruit fly *Drosophila melanogaster* unfolds the secrets of innate immunity. Acta Paediatr.

[7] Castillo JC, Reynolds SE, Eleftherianos I (2011). Insect immune responses to nematode parasites. Trends Parasitol.

[8] Dillman AR, Chaston JM, Adams BJ, Ciche TA, Goodrich-Blair H, Stock SP (2012). An entomopathogenic nematode by any other name. PLoS Pathog.

[9] Ciche TA, Darby C, Ehlers RU, Forst S, Goodrich-Blair H (2006). Dangerous liaisons: The symbiosis of entomopathogenic nematodes and bacteria. Biol Control.

[10] Ciche T. The biology and genome of *Heterorhabditis bacteriophora*. WormBook. 2007;1–9. 10.1895/wormbook.1.135.1 PMC4781484 18050499

[11] Ffrench-Constant RH, Dowling A, Waterfield NR (2007). Insecticidal toxins from *Photorhabdus* bacteria and their potential use in agriculture. Toxicon.

[12] Bode HB (2009). Entomopathogenic bacteria as a source of secondary metabolites. Curr Opin Chem Biol.

[13] Ciche TA, Ensign JC (2003). For the insect pathogen *Photorhabdus luminescens*, which end of a nematode is out?. Appl Environ Microbiol.

[14] Hallem EA, Rengarajan M, Ciche TA, Sternberg PW (2007). Nematodes, bacteria, and flies: a tripartite model for nematode parasitism. Curr Biol.

[15] Wang Z, Wilhelmsson C, Hyrsl P, Loof TG, Dobes P, Klupp M (2010). Pathogen entrapment by transglutaminase–a conserved early innate immune mechanism. PLOS Pathog.

[16] Hyrsl P, Dobes P, Wang Z, Hauling T, Wilhelmsson C, Theopold U (2011). Clotting factors and eicosanoids protect against nematode infections. J Innate Immun.

[17] Castillo JC, Shokal U, Eleftherianos I (2013). Immune gene transcription in *Drosophila* adult flies infected by entomopathogenic nematodes and their mutualistic bacteria. J Insect Physiol.

[18] Arefin B, Kucerova L, Dobes P, Markus R, Strnad H, Wang Z (2014). Genome-wide transcriptional analysis of *Drosophila* larvae infected by entomopathogenic nematodes shows involvement of complement, recognition and extracellular matrix proteins. J Innate Immun.

[19] Wang Z, Gerstein M, Snyder M (2009). RNA-Seq: a revolutionary tool for transcriptomics. Nat Rev Genet.

[20] Ozsolak F, Milos PM (2011). RNA sequencing: advances, challenges and opportunities. Nat Rev Genet.

[21] de Klerk E, den Dunnen JT, 't Hoen PA (2014). RNA sequencing: from tag-based profiling to resolving complete transcript structure. Cell Mol Life Sci.

[22] Xuan J, Yu Y, Qing T, Guo L, Shi L (2013). Next-generation sequencing in the clinic: promises and challenges. Cancer Lett.

[23] Costa V, Aprile M, Esposito R, Ciccodicola A (2013). RNA-Seq and human complex diseases: recent accomplishments and future perspectives. Eur J Hum Genet.

[24] Daines B, Wang H, Wang L, Li Y, Han Y, Emmert D (2011). The *Drosophila melanogaster* transcriptome by paired-end RNA sequencing. Genome Res.

[25] Ekblom R, Galindo J (2011). Applications of next generation sequencing in molecular ecology of non-model organisms. Heredity.

[26] Westermann AJ, Gorski SA, Vogel J (2012). Dual RNA-seq of pathogen and host. Nat Rev Microbiol.

[27] Castillo JC, Shokal U, Eleftherianos I (2012). A novel method for infecting *Drosophila* adult flies with insect pathogenic nematodes. Virulence.

[28] Trapnell C, Roberts A, Goff L, Pertea G, Kim D, Kelley DR (2012). Differential gene and transcript expression analysis of RNA-seq experiments with TopHat and Cufflinks. Nat Protoc.

[29] Kanehisa M, Goto S, Kawashima S, Okuno Y, Hattori M (2004). The KEGG resource for deciphering the genome. Nucleic Acids Res.

[30] Huang DW, Sherman BT, Lempicki RA (2009). Systematic and integrative analysis of large gene lists using DAVID Bioinformatics Resources. Nat Protoc.

[31] Huang DW, Sherman BT, Lempicki RA (2009). Bioinformatics enrichment tools: paths toward the comprehensive functional analysis of large gene lists. Nucleic Acids Res.

[32] Mi H, Thomas P (2009). PANTHER pathway: an ontology-based pathway database coupled with data analysis tools. Methods Mol Biol.

[33] Thomas PD, Mi H, Lewis S (2007). Ontology annotation: mapping genomic regions to biological function. Curr Opin Chem Biol.

[34] Ayres JS, Freitag N, Schneider DS (2008). Identification of *Drosophila* mutants altering defense of and endurance to *Listeria monocytogenes* infection. Genetics.

[35] Özkan E, Carrillo RA, Eastman CL, Weiszmann R, Waghray D, Johnson KG (2013). An extracellular interactome of immunoglobulin and LRR proteins reveals receptor-ligand networks. Cell.

[36] Gummalla M, Maeda RK, Castro Alvarez JJ, Gyurkovics H, Singari S, Edwards KA (2012). abd-A regulation by the iab-8 noncoding RNA. PLoS Genet.

[37] Wright VM, Vogt KL, Smythe E, Zeidler MP (2011). Differential activities of the *Drosophila* JAK/STAT pathway ligands Upd, Upd2 and Upd3. Cell Signal.

[38] Ekengren S, Hultmark D (2001). A family of *Turandot*-related genes in the humoral stress response of *Drosophila*. Biochem Biophys Res Commun.

[39] Brun S, Vidal S, Spellman P, Takahashi K, Tricoire H, Lemaitre B (2006). The MAPKKK Mekk1 regulates the expression of *Turandot* stress genes in response to septic injury in *Drosophila*. Genes Cells.

[40] Gordon MD, Ayres JS, Schneider DS, Nusse R (2008). Pathogenesis of listeria-infected *Drosophila wntD* mutants is associated with elevated levels of the novel immunity gene *edin*. PLoS Pathog.

[41] Mellroth P, Karlsson J, Steiner HA (2003). Scavenger function for a Drosophila peptidoglycan recognition protein. J Biol Chem.

[42] Tanji T, Ohashi-Kobayashi A, Natori S (2006). Participation of a galactose-specific C-type lectin in *Drosophila* immunity. Biochem J.

[43] Stroschein-Stevenson SL, Foley E, O'Farrell PH, Johnson AD (2006). Identification of *Drosophila* gene products required for phagocytosis of *Candida albicans*. PLoS Biol.

[44] Karouzou MV, Spyropoulos Y, Iconomidou VA, Cornman RS, Hamodrakas SJ, Willis JH (2007). *Drosophila* cuticular proteins with the R&R Consensus: annotation and classification with a new tool for discriminating RR-1 and RR-2 sequences. Insect Biochem Mol Biol.

[45] Perrimon N, Smouse D, Miklos GLG (1989). Developmental genetics of loci at the base of the X chromosome of *Drosophila melanogaster*. Genetics..

[46] Kambris Z, Brun S, Jang IH, Nam HJ, Romeo Y, Takahashi K (2006). *Drosophila* immunity: a large-scale *in vivo* RNAi screen identifies five serine proteases required for Toll activation. Curr Biol.

[47] Chakrabarti S, Poidevin M, Lemaitre B (2014). The *Drosophila* MAPK p38c Regulates Oxidative Stress and Lipid Homeostasis in the Intestine. PLoS Genet.

[48] Varghese J, Lim SF, Cohen SM (2010). *Drosophila* miR-14 regulates insulin production and metabolism through its target, *sugarbabe*. Genes Dev.

[49] Tang AH, Tu CPD (1994). Biochemical characterization of *Drosophila* glutathione S-transferases D1 and D21. J Biol Chem.

[50] Han Q, Fang J, Ding H, Johnson JK, Christensen BM, Li J (2002). Identification of *Drosophila melanogaster* yellow-f and yellow-f2 proteins as dopachrome-conversion enzymes. Biochem J.

[51] Clyne PJ, Warr CG, Carlson JR (2000). Candidate taste receptors in *Drosophila*. Science.

[52] Andrés M, Turiégano E, Göpfert MC, Canal I, Torroja L (2014). The extracellular matrix protein artichoke is required for integrity of ciliated mechanosensory and chemosensory organs in *Drosophila* embryos. Genetics.

[53] Sieber MH, Thummel CS (2012). Coordination of Triacylglycerol and Cholesterol Homeostasis by DHR96 and the *Drosophila* LipA Homolog magro. Cell Metab.

[54] Kleino A, Silverman N (2014). The *Drosophila* IMD pathway in the activation of the humoral immune response. Dev Comp Immunol.

[55] Lindsay SA, Wasserman SA (2014). Conventional and non-conventional *Drosophila* Toll signaling. Dev Comp Immunol.

[56] Myllymäki H, Rämet M (2014). Jak/STAT pathway in *Drosophila* immunity. Scand J Immunol.

[57] Blandin S, Levashina EA (2004). Thioester-containing proteins and insect immunity. Mol Immunol.

[58] Delaney JR, Stoven S, Uvell H, Anderson KV, Engstrom Y, Mlodzik M (2006). Cooperative control of *Drosophila* immune responses by the JNK and NF-kappaB signaling pathways. EMBO J.

[59] Ríos-Barrera LD, Riesgo-Escovar JR (2013). Regulating cell morphogenesis: the *Drosophila* Jun N-terminal kinase pathway. Genesis.

[60] Hamilton PT, Leong JS, Koop BF, Perlman SJ (2014). Transcriptional responses in a *Drosophila* defensive symbiosis. Mol Ecol.

[61] Krishnan N, Dickman MB, Becker DF (2008). Proline modulates the intracellular redox environment and protects mammalian cells against oxidative stress. Free Radic Biol Med.

[62] Phang JM, Liu W (2012). Proline metabolism and cancer. Front Biosci.

[63] McIntire CR, Yeretssian G, Saleh M (2009). Inflammasomes in infection and inflammation. Apoptosis.

[64] Ffrench-Constant R, Waterfield N, Daborn P, Joyce S, Bennett H, Au C (2003). *Photorhabdus*: towards a functional genomic analysis of a symbiont and pathogen. FEMS Microbiol Rev.

[65] Irving P, Troxler L, Heuer TS, Belvin M, Kopczynski C, Reichhart JM (2001). A genome-wide analysis of immune responses in *Drosophila*. Proc Natl Acad Sci USA.

[66] De Gregorio E, Spellman PT, Tzou P, Rubin GM, Lemaitre B (2002). The Toll and Imd pathways are the major regulators of the immune response in *Drosophila*. EMBO J.

[67] Apidianakis Y, Mindrinos MN, Xiao W, Lau GW, Baldini RL, Davis RW (2005). Profiling early infection responses: *Pseudomonas aeruginosa* eludes host defenses by suppressing antimicrobial peptide gene expression. Proc Natl Acad Sci USA.

[68] Sonnleitner E, Valentini M, Wenner N, Haichar FZ, Haas D, Lapouge K (2012). Novel targets of the CbrAB/Crc carbon catabolite control system revealed by transcript abundance in *Pseudomonas aeruginosa*. PLoS One.

[69] Dhur A, Galan P, Hercberg S (1991). Folate status and the immune system. Prog Food Nutr Sci.

[70] Courtemanche C, Elson-Schwab I, Mashiyama ST, Kerry N, Ames BN (2004). Folate deficiency inhibits the proliferation of primary human CD8+ T lymphocytes *in vitro*. J Immunol.

[71] Silva CP, Waterfield NR, Daborn PJ, Dean P, Chilver T, Au CP (2002). Bacterial infection of a model insect: Photorhabdus luminescens and Manduca sexta. Cell Microbiol.

[72] Eleftherianos I, Ffrench-Constant RH, Clarke DJ, Dowling AJ, Reynolds SE (2010). Dissecting the immune response to the entomopathogen *Photorhabdus*. Trends Microbiol.

[73] Royet J (2011). Epithelial homeostasis and the underlying molecular mechanisms in the gut of the insect model *Drosophila melanogaster*. Cell Mol Life Sci.

[74] Davis MM, Engström Y (2012). Immune response in the barrier epithelia: lessons from the fruit fly *Drosophila melanogaster*. J Innate Immun.

[75] Kuraishi T, Hori A, Kurata S (2013). Host-microbe interactions in the gut of *Drosophila melanogaster*. Front Physiol.

[76] Imler JL, Bulet P (2005). Antimicrobial peptides in *Drosophila*: structures, activities and gene regulation. Chem Immunol Allergy.

[77] Lemaitre B, Hoffmann J (2007). The host defense of *Drosophila melanogaster*. Ann Rev Immunol.

[78] Blandin SA, Marois E, Levashina EA (2008). Antimalarial responses in *Anopheles gambiae*: from a complement-like protein to a complement-like pathway. Cell Host Microbe.

[79] Bou Aoun R, Hetru C, Troxler L, Doucet D, Ferrandon D, Matt N (2011). Analysis of thioester-containing proteins during the innate immune response of *Drosophila melanogaster*. J Innate Immun.

[80] Thompson AA, Binham J, Plant T, Whyte MK, Walmsley SR (2013). Hypoxia, the HIF pathway and neutrophilic inflammatory responses. Biol Chem.

[81] Gorr TA, Wichmann D, Hu J, Hermes-Lima M, Welker AF, Terwilliger N (2010). Hypoxia tolerance in animals: biology and application. Physiol Biochem Zool.

[82] Hasnain SZ, Gallagher AL, Grencis RK, Thornton DJ (2012). A new role for mucins in immunity: insights from gastrointestinal nematode infection. Int J Biochem Cell Biol.

[83] Fundytus ME (2001). Glutamate receptors and nociception: implications for the drug treatment of pain. CNS Drugs.

[84] Szekely JI, Torok K, Mate G (2002). The role of ionotropic glutamate receptors in nociception with special regard to the AMPA binding sites. Curr Pharm Des.

[85] Numazaki M, Tominaga M (2004). Nociception and TRP Channels. Curr Drug Targets CNS Neurol Disord.

[86] Benton R, Vannice KS, Gomez-Diaz C, Vosshall LB (2009). Variant ionotropic glutamate receptors as chemosensory receptors in *Drosophila*. Cell.

[87] Robertson JL, Tsubouchi A, Tracey WD (2013). Larval defense against attack from parasitoid wasps requires nociceptive neurons. PLoS One.

[88] AbuHatab M, Selvan S, Gaugler R (1995). Role of proteases in penetration of insect gut by the entomopathogenic nematode *Steinernema glaseri* (Nematoda: Steinernematidae). J Invert Path.

[89] McKerrow JH, Caffrey C, Kelly B, Loke P, Sajid M (2006). Proteases in parasitic diseases. Annu Rev Pathol.

[90] Bai X, Adams BJ, Ciche TA, Clifton S, Gaugler R, Kim KS (2013). A lover and a fighter: the genome sequence of an entomopathogenic nematode *Heterorhabditis bacteriophora*. PLoS One.

[91] Babusyte A, Kotthoff M, Fiedler J, Krautwurst D (2013). Biogenic amines activate blood leukocytes via trace amine-associated receptors TAAR1 and TAAR2. J Leukoc Biol.

[92] Ligoxygakis P (2013). Genetics of immune recognition and response in *Drosophila* host defense. Adv Genet.

[93] Cornman RS (2009). Molecular evolution of *Drosophila* cuticular protein genes. PLoS One.

[94] Rebers JE, Willis JH (2001). A conserved domain in arthropod cuticular proteins binds chitin. Insect Biochem Mol Biol.

[95] Cornman RS, Willis JH (2009). Annotation and analysis of low-complexity protein families of *Anopheles gambiae* that are associated with cuticle. Insect Mol Biol.

[96] Tang L, Liang J, Zhan Z, Xiang Z, He N (2010). Identification of the chitin-binding proteins from the larval proteins of silkworm, Bombyx mori. Insect Biochem Mol Biol.

[97] Gendrin M, Zaidman-Rémy A, Broderick NA, Paredes J, Poidevin M, Roussel A (2013). Functional analysis of PGRP-LA in *Drosophila* immunity. PLoS One.

[98] White GFR (1927). A method for obtaining infective nematode larvae from cultures. Science.

[99] Trapnell C, Pachter L, Salzberg SL (2009). TopHat: discovering splice junctions with RNA-Seq. Bioinformatics.

[100] Langmead B. Aligning short sequencing reads with Bowtie. Curr Protoc Bioinformatics. 2010, Chapter 11:Unit 11.7. 10.1002/0471250953.bi1107s32 PMC3010897 21154709

[101] Aanes H, Winata C, Moen LF, Østrup O, Mathavan S, Collas P (2014). Normalization of RNA-sequencing data from samples with varying mRNA levels. PLoS One.

