# Supplementary material for: Drosophila anti-nematode and antibacterial immune regulators revealed by RNA-Seq
Source: BMC Genomics. 2015 Jul 11;16(1):519. doi: 10.1186/s12864-015-1690-2 (PMC4499211; doi:10.1186/s12864-015-1690-2)
Supplement: Additional file 1: — Text description. Figure S1. Gene coverage density plot. Figure S2. Quantitative real-time RT-PCR validation. Figure S3. Infection of Drosophila flies with Heterorhabditis nematodes or their Photorhabdus bacteria induces diverse physiological responses. Figure S4. Quadrant plots showing expression patterns in adult flies infected by nematodes or their bacteria. Figure S5. Infection of Drosophila flies with Heterorhabditis or their Photorhabdus suppresses the expression of several genes. Figure S6. Gene Ontology analysis. Table S1. List of primers used for quantitative real-time RT-PCR validation. [file 12864_2015_1690_MOESM1_ESM.pdf]

Supplementary figure fegends

Supplementary Figure 1 Gene coverage density plot. Representation of the gene coverage generated by the sequenced reads that were obtained for each experimental condition: infection of *Drosophila melanogaster* wild-type adult flies with *Heterorhabditis* axenic nematodes, *Heterorhabditis* symbiotic nematodes, *Photorhabdus* bacteria at 12 h and 30 h post-infection, and uninfected control.

Supplementary Figure 2 Quantitative real-time RT-PCR validation. Seven *Drosophila melanogaster* genes (*CG34040*, *CG64267*, *CG9468*, *CG11909*, *CG6524*, *CG17571*, *CG10374*) were selected from the RNAseq dataset and qRT-PCR results are shown from flies infected by *Heterorhabditis bacteriophora* symbiotic nematodes for 12 h. Solid bars represent transcript levels obtained from RNAseq; open bars show expression levels obtained from qRT-PCR experiments. Flies used for RNAseq and qRT-PCR assays were subjected to the same experimental conditions.

Supplementary Figure 3 Infection of *Drosophila* flies with *Heterorhabditis* nematodes or their *Photorhabdus* bacteria induces diverse physiological responses. Enrichment of pathway specific genes according to the KEGG and PANTHER classification databases. Representative KEGG pathway categories in flies infected by **(A)** axenic *Heterorhabditis* nematodes, **(B)** symbiotic *Heterorhabditis* nematodes, and **(C)** *Photorhabdus* bacteria at 12 h post-infection. The number of genes represents those that were only found associated with a particular pathway. Representative PANTHER pathway categories in flies infected with **(D)** axenic *Heterorhabditis* nematodes, **(E)** symbiotic *Heterorhabditis* nematodes, and **(F)** *Photorhabdus* bacteria at 30 h post-infection.

Supplemental Figure 4 Quadrant plots showing expression patterns in adult flies infected by nematodes or their bacteria. Upper right (UP/UP): genes up-regulated upon infection; Upper left (DOWN/UP): genes up-regulated at 30 h only; Lower right (UP/DOWN): genes up-regulated at 12 h only; Lower left (DOWN/DOWN): genes down-regulated upon infection.

Supplementary Figure 5 Infection of *Drosophila* flies with *Heterorhabditis* or their *Photorhabdus* suppresses the expression of several genes. The 25 most strongly downregulated genes upon infection with **(A)** axenic *Heterorhabditis* nematodes **(B)** symbiotic *Heterorhabditis* nematodes and **(C)** *Photorhabdus* bacteria. X-axis represents the relative Log-Fold Change (LFC) for each gene after normalization against uninfected controls. All genes have a fold change higher than 2 (LFC=0.58 corresponds to 2-fold change difference).

Supplementary Figure 6 Gene Ontology analysis. GO immune response categories identified in *Drosophila melanogaster* wild-type adult flies infected by **(A)** axenic *Heterorhabditis* nematodes, **(B)** symbiotic *Heterorhabditis* nematodes, or **(C)** *Photorhabdus* bacteria at 12 h post-infection. The Y-axis corresponds to the number of genes for each GO category and their relative level of expression (upregulation or down-regulation). All genes have a fold change higher than 2 (Log-Fold Change=0.58 corresponds to 2-fold change difference).

Supplementary Fig. S1

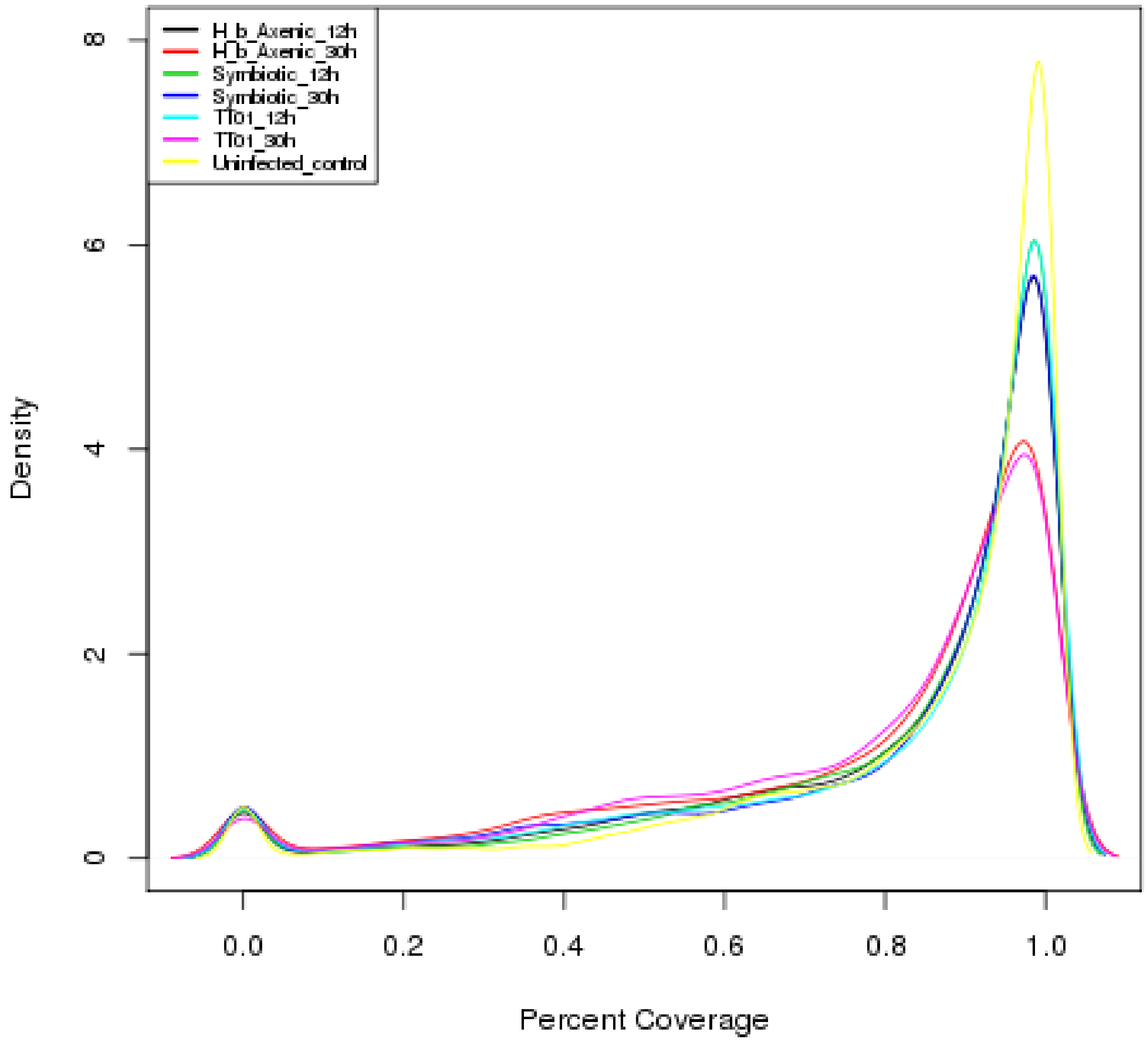

Supplementary Fig. S2

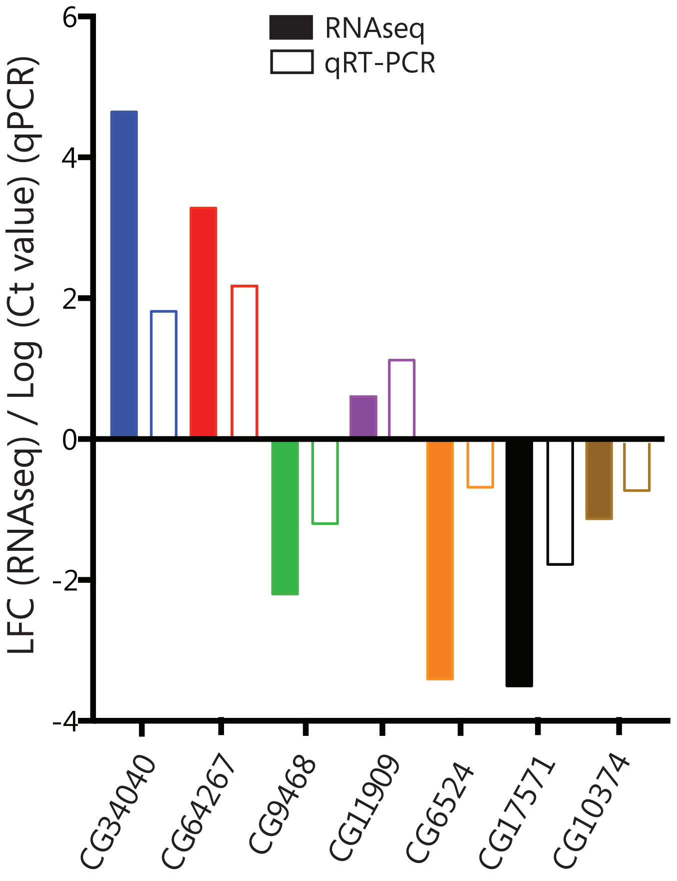

Supplementary Fig. S3

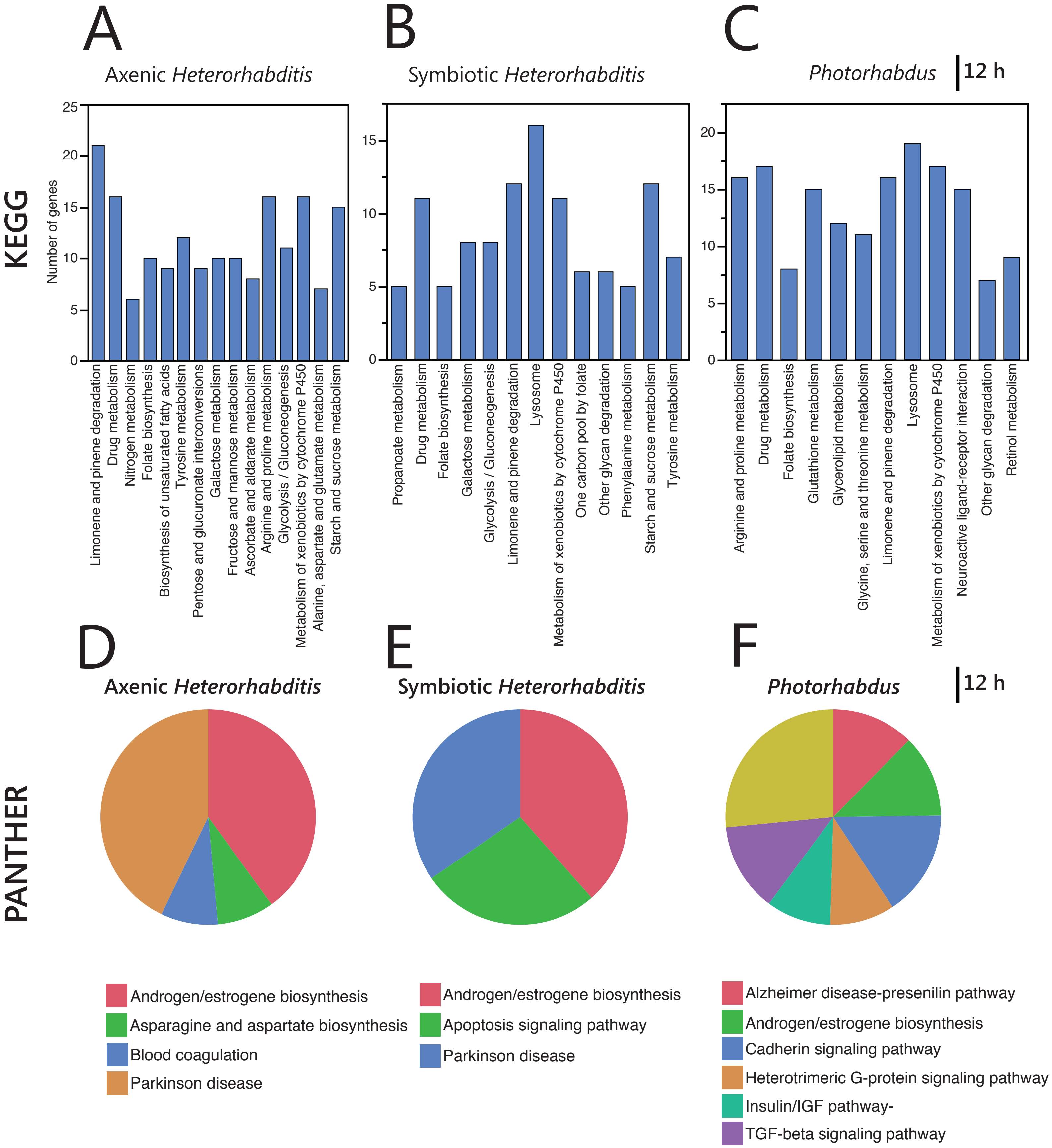

Supplementary Fig. S4

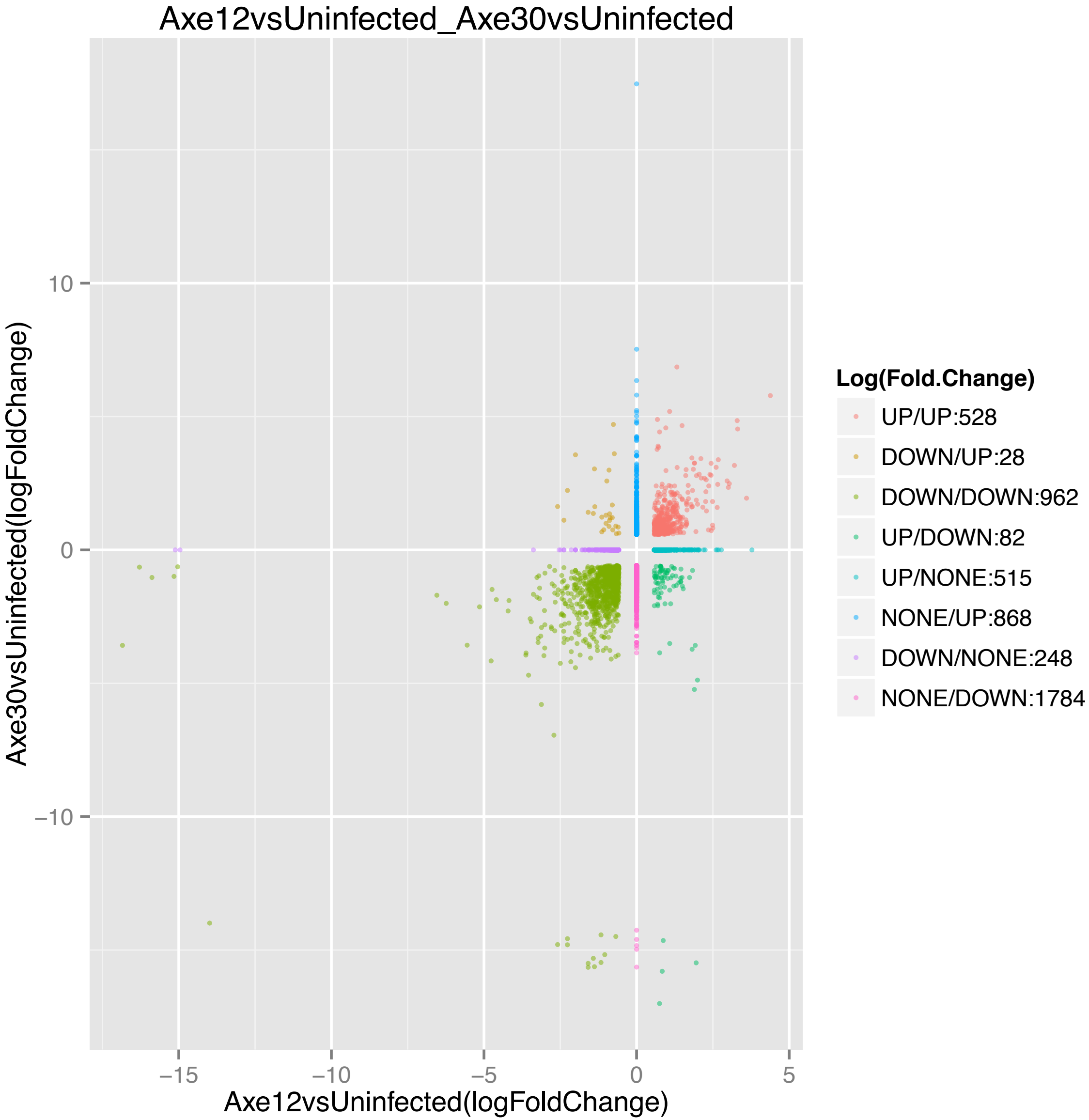

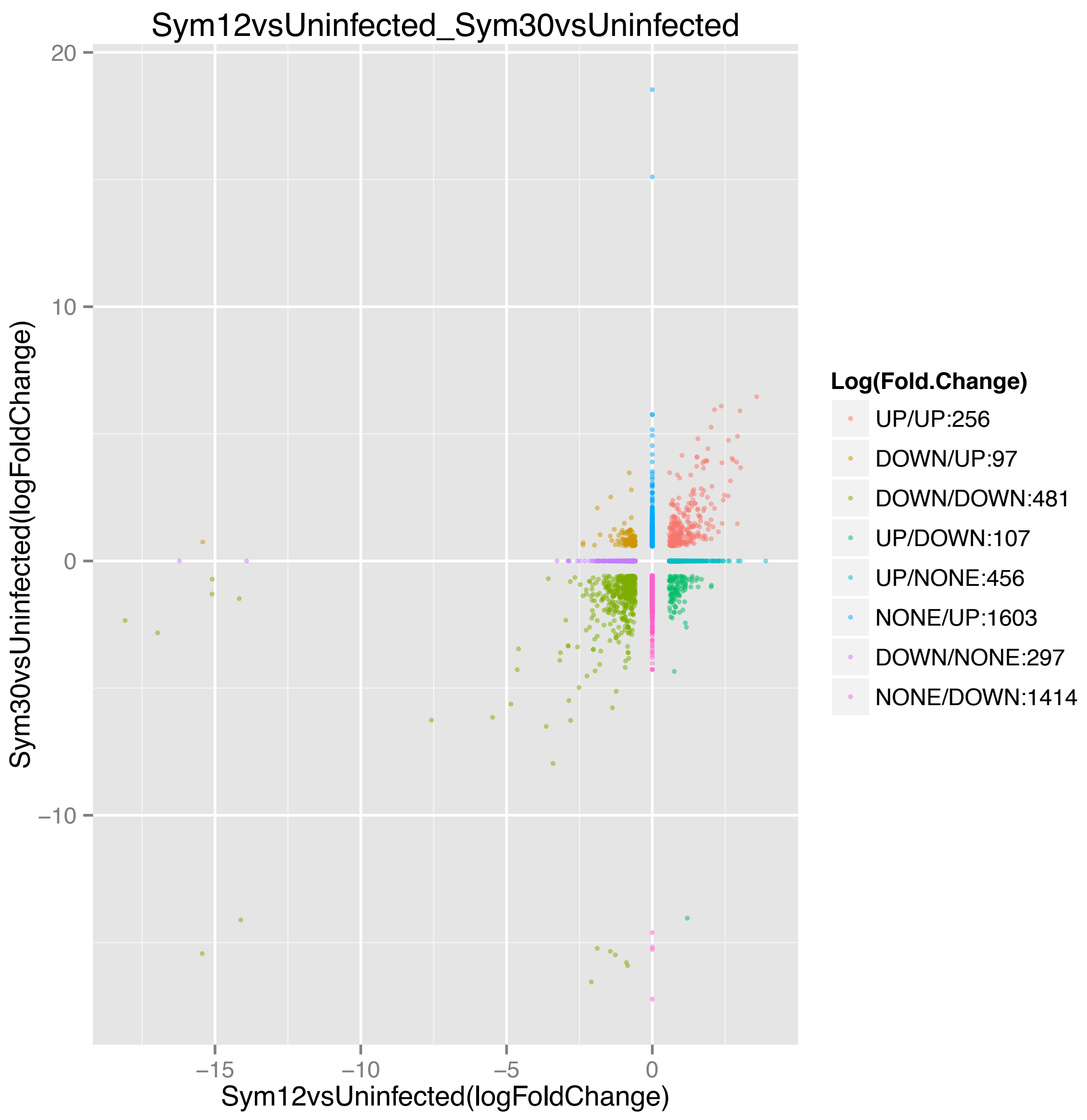

Sym12vsUninfected_Sym30vsUninfected
Sym30vsUninfected(logFoldChange)
Sym12vsUninfected(logFoldChange)
20
10
0
−10
−15
−10
−5
0
Log(Fold.Change)
UP/UP:256
DOWN/UP:97
DOWN/DOWN:481
UP/DOWN:107
UP/NONE:456
NONE/UP:1603
DOWN/NONE:297
NONE/DOWN:1414

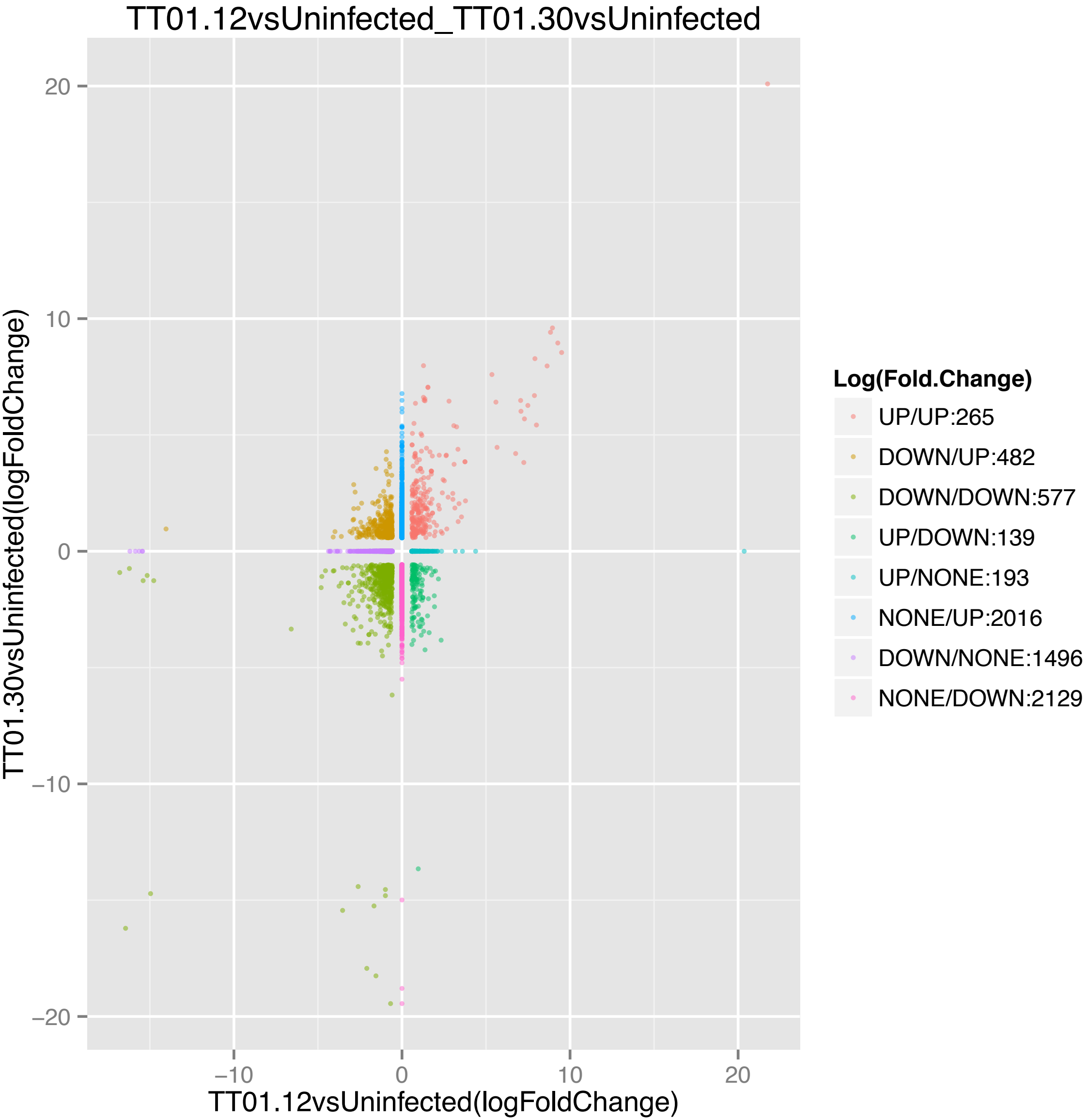

TT01.12vsUninfected_TT01.30vsUninfected
20
10
0
−10
−20
−10
0
10
20
TT01.12vsUninfected(logFoldChange)
TT01.30vsUninfected(logFoldChange)
Log(Fold.Change)
UP/UP:265
DOWN/UP:482
DOWN/DOWN:577
UP/DOWN:139
UP/NONE:193
NONE/UP:2016
DOWN/NONE:1496
NONE/DOWN:2129

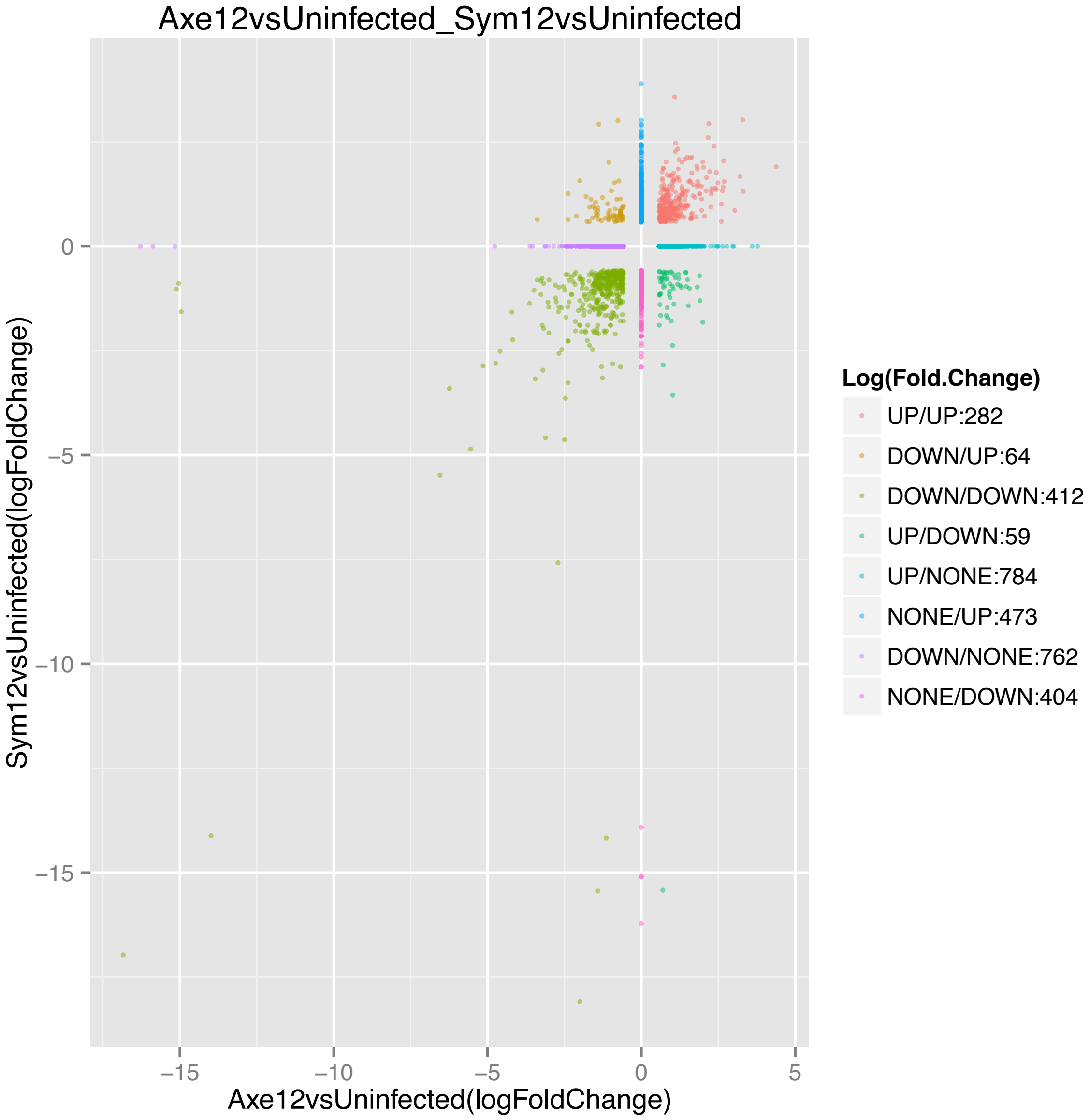

Axe12vsUninfected_Sym12vsUninfected
Sym12vsUninfected(logFoldChange)
Axe12vsUninfected(logFoldChange)
0
−5
−10
−15
5
Log(Fold.Change)
UP/UP:282
DOWN/UP:64
DOWN/DOWN:412
UP/DOWN:59
UP/NONE:784
NONE/UP:473
DOWN/NONE:762
NONE/DOWN:404

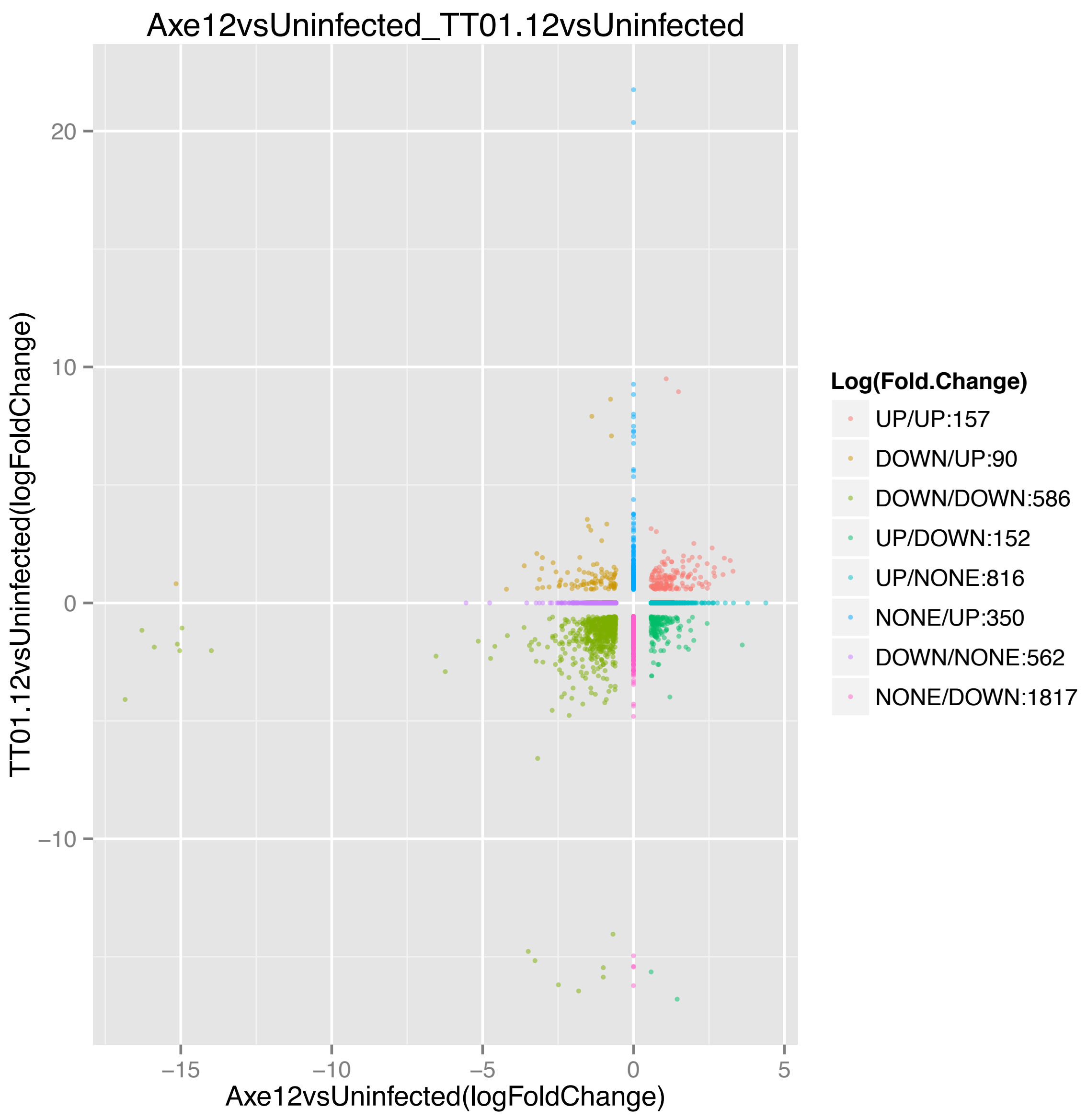
Axe12vsUninfected_TT01.12vsUninfected
TT01.12vsUninfected(logFoldChange)
Axe12vsUninfected(logFoldChange)
20
10
0
−10
−15
−10
−5
0
5
Log(Fold.Change)
UP/UP:157
DOWN/UP:90
DOWN/DOWN:586
UP/DOWN:152
UP/NONE:816
NONE/UP:350
DOWN/NONE:562
NONE/DOWN:1817

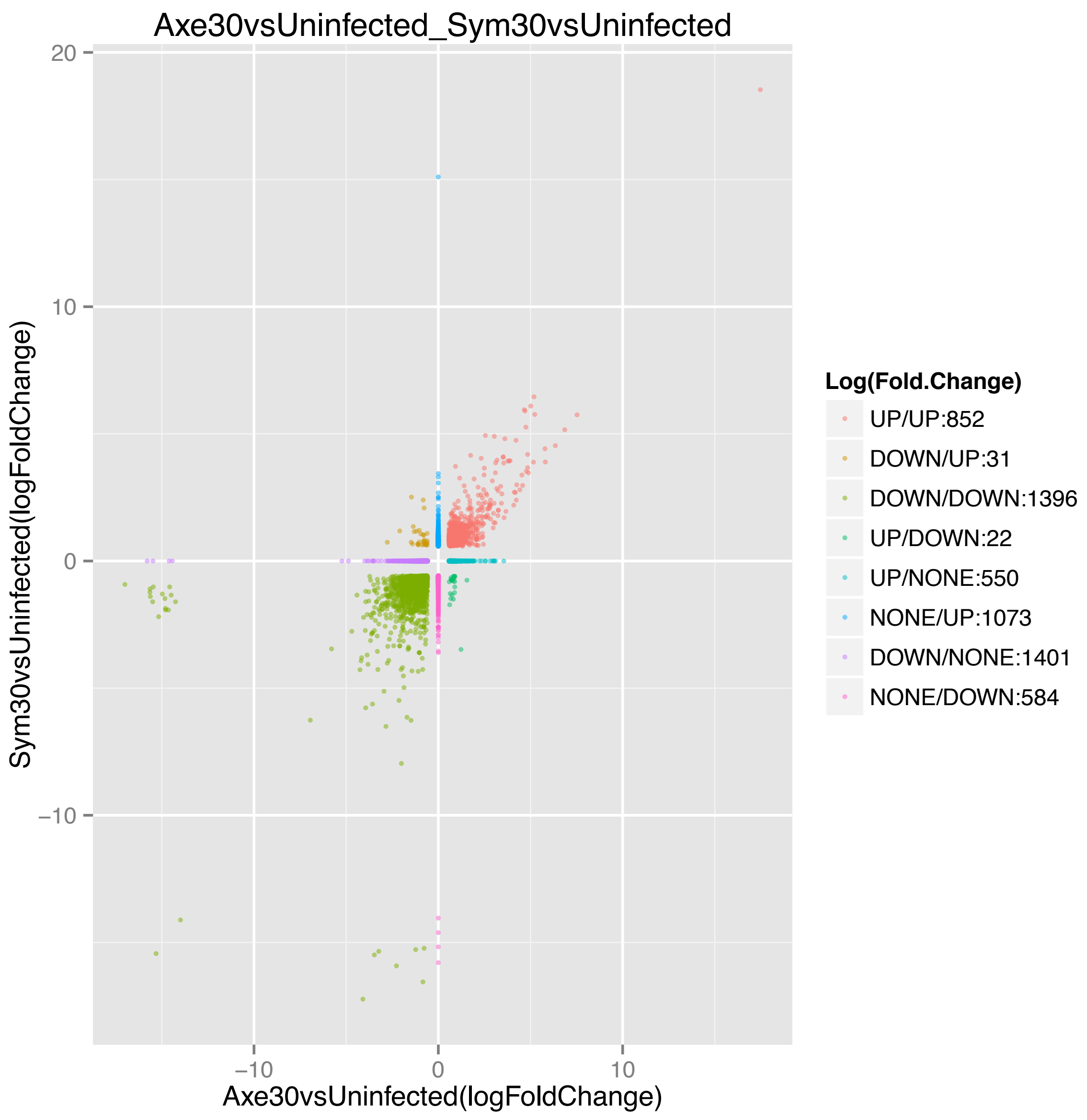

Axe30vsUninfected_Sym30vsUninfected
Sym30vsUninfected(logFoldChange)
Axe30vsUninfected(logFoldChange)
20
10
0
−10
−10
0
10
Log(Fold.Change)
UP/UP:852
DOWN/UP:31
DOWN/DOWN:1396
UP/DOWN:22
UP/NONE:550
NONE/UP:1073
DOWN/NONE:1401
NONE/DOWN:584

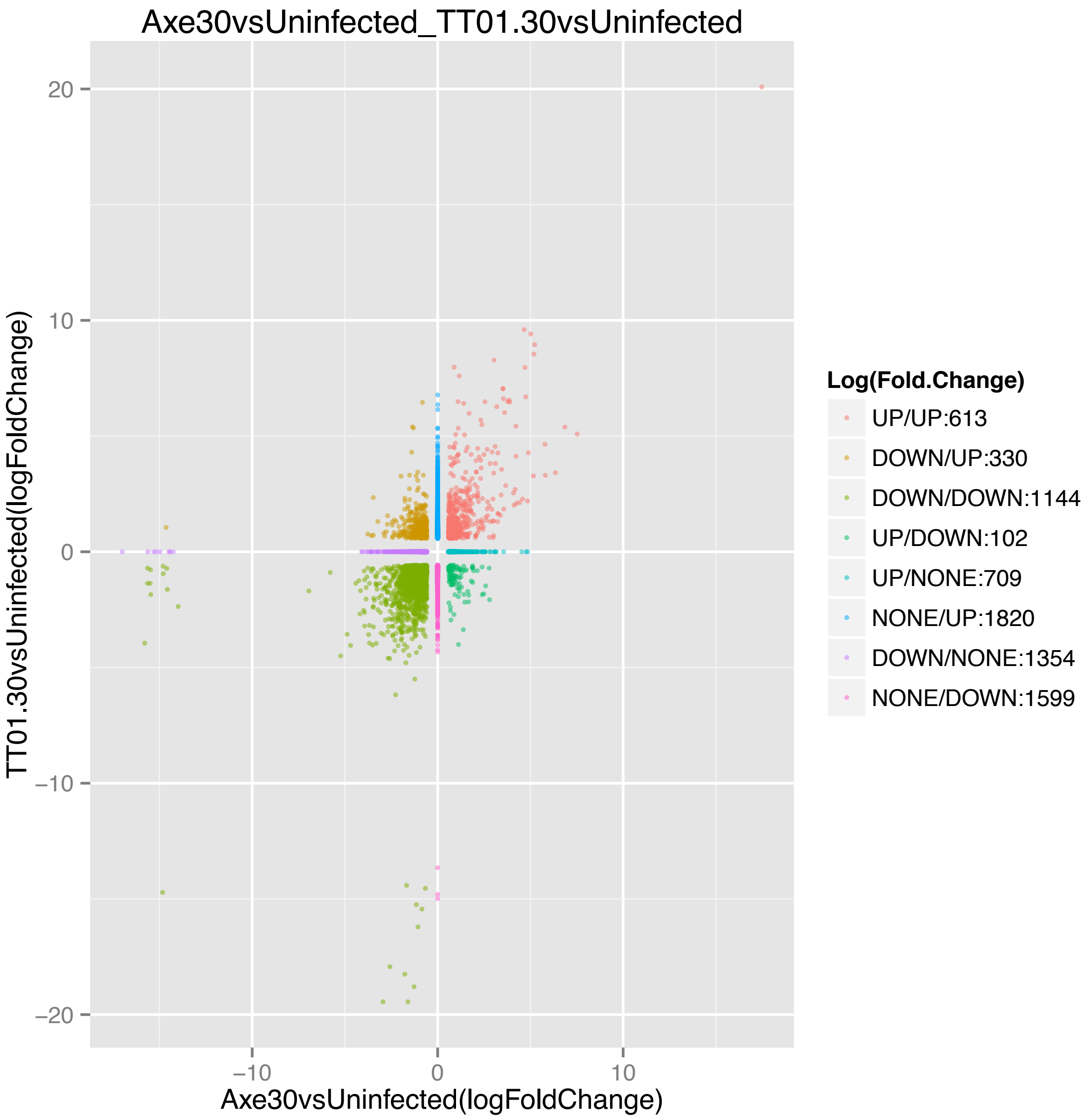

Axe30vsUninfected_TT01.30vsUninfected
20
10
0
−10
−20
TT01.30vsUninfected(logFoldChange)
−10
0
10
Axe30vsUninfected(logFoldChange)
Log(Fold.Change)
UP/UP:613
DOWN/UP:330
DOWN/DOWN:1144
UP/DOWN:102
UP/NONE:709
NONE/UP:1820
DOWN/NONE:1354
NONE/DOWN:1599

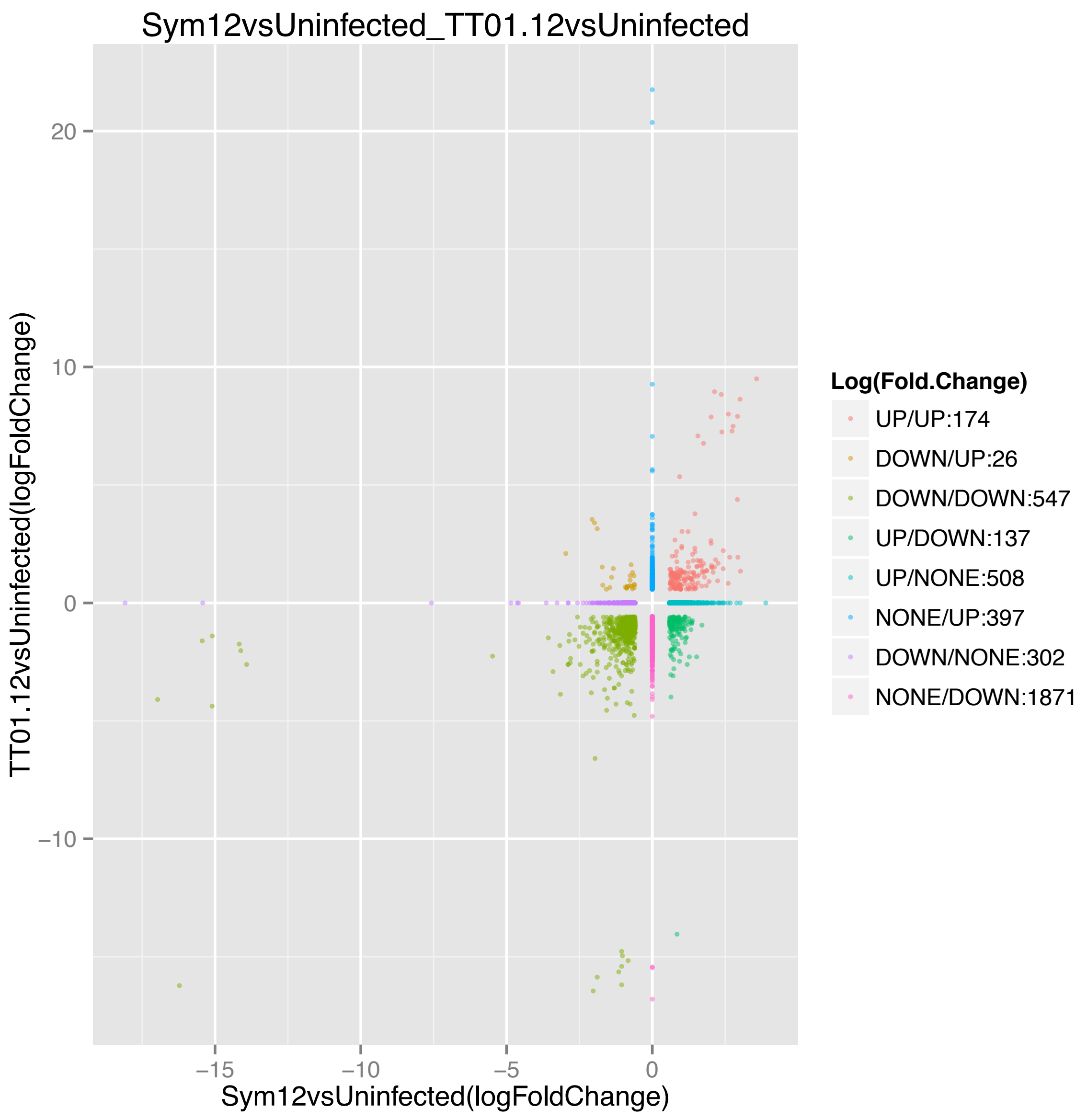

Sym12vsUninfected_TT01.12vsUninfected
20
10
0
−10
TT01.12vsUninfected(logFoldChange)
−15
−10
−5
0
Sym12vsUninfected(logFoldChange)
Log(Fold.Change)
UP/UP:174
DOWN/UP:26
DOWN/DOWN:547
UP/DOWN:137
UP/NONE:508
NONE/UP:397
DOWN/NONE:302
NONE/DOWN:1871

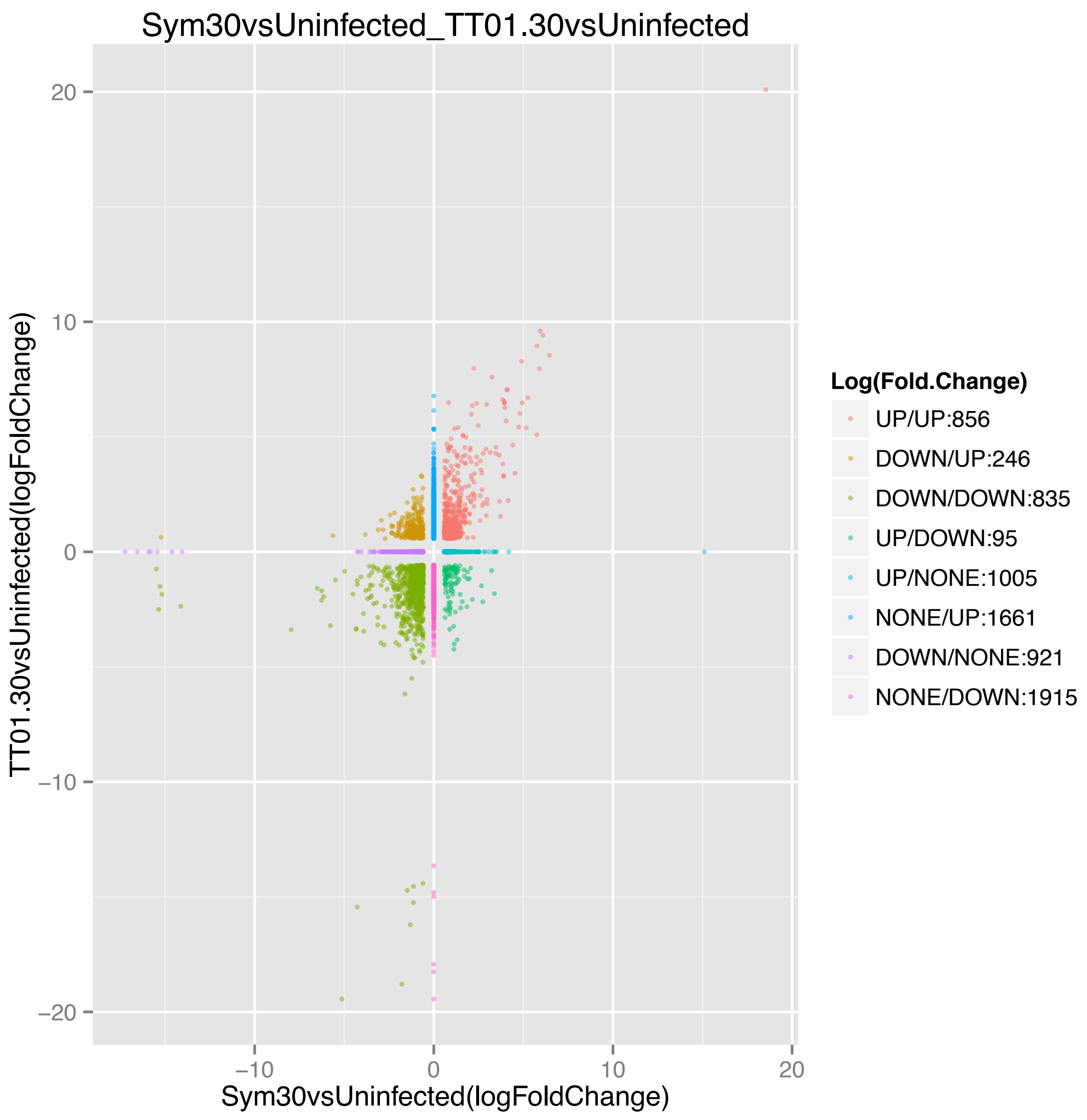
Sym30vsUninfected_TT01.30vsUninfected
TT01.30vsUninfected(logFoldChange)
Sym30vsUninfected(logFoldChange)
20
10
0
−10
−20
−10
0
10
20
Log(Fold.Change)
UP/UP:856
DOWN/UP:246
DOWN/DOWN:835
UP/DOWN:95
UP/NONE:1005
NONE/UP:1661
DOWN/NONE:921
NONE/DOWN:1915

Supplementary Fig. S5

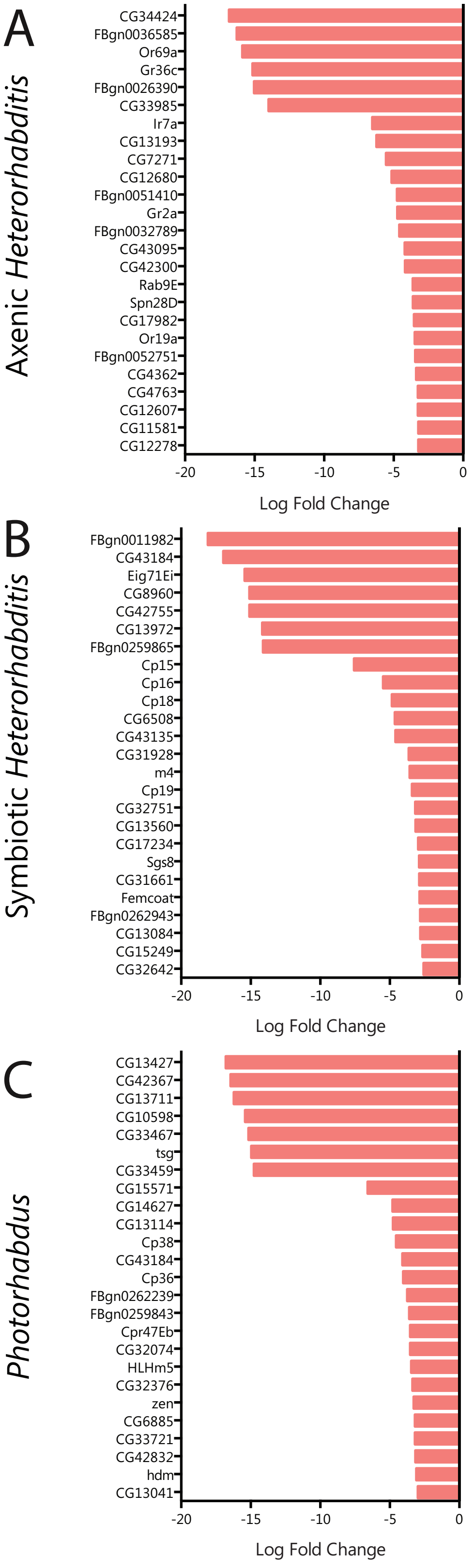

Supplementary Fig. S6

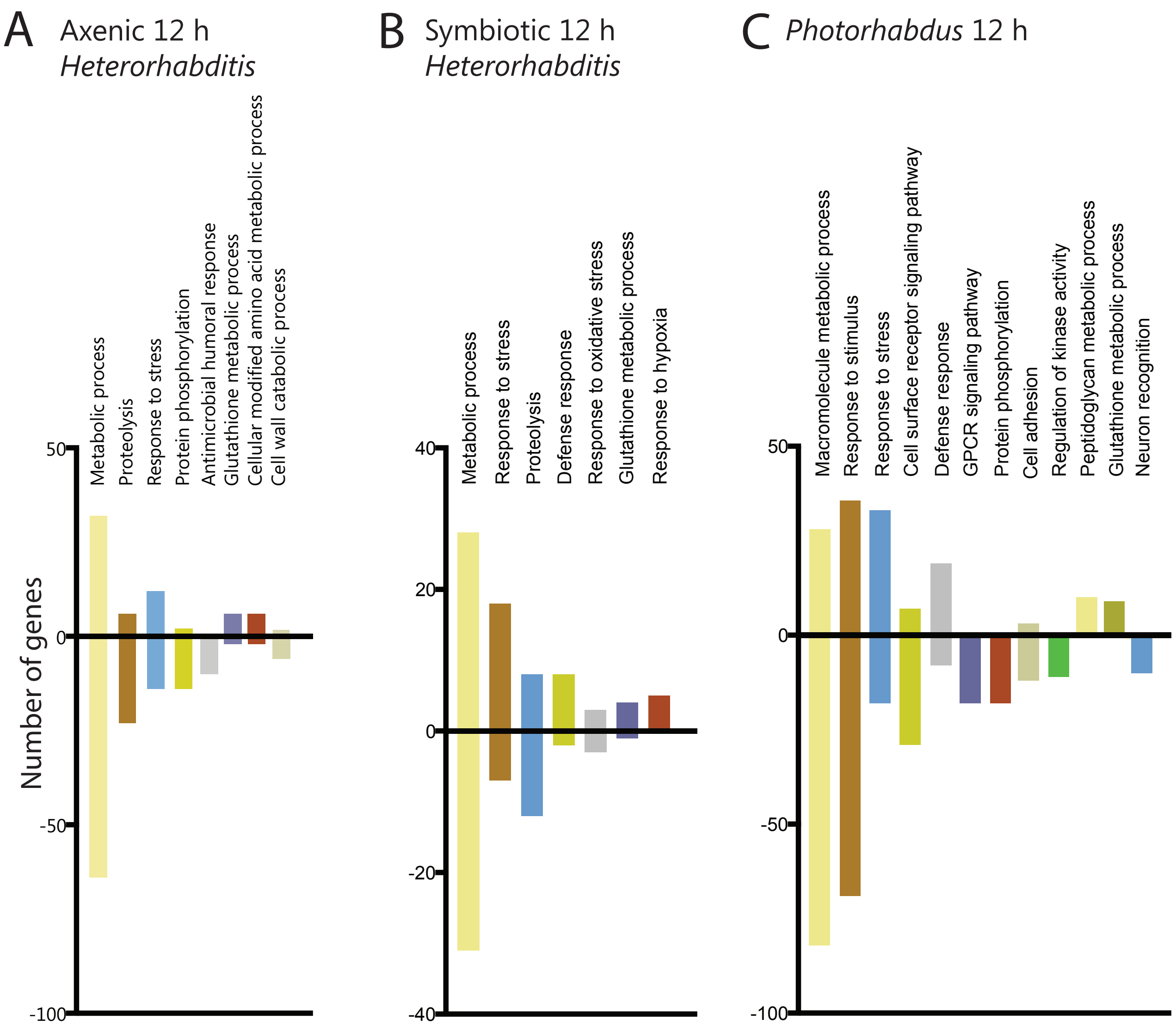

Supplementary Table 1 List of primers used for quantitative real-time RT-PCR validation.

Gene Name	Accession no	Comments	Sequence
**Unknown**	CG34040	Unknown function DUF753	Forward 5'-TCGGGATATAAACTGCCAAAGGAG-3'<br>Reverse 5'-GTTAGTAGCCCGGGAGATTCAGC-3'
**Unknown**	CG64267	Unknown function	Forward 5'-TGTTCCGCATTATCGCTGTGA-3'<br>Reverse 5'-AAAATTTAACCGTGGTAGATGTGG-3'
**Unknown**	CG9468	Glycoside hydrolase family	Forward 5'-GCCCGTCCCCGCATTGGTT-3'<br>Reverse 5'-CCGCAGTGGACATCGAAGCAGTAG-3'
Tobi	CG11909	Aldolase-type TIM barrel; Glycoside hydrolase	Forward 5'-TGCAGGCGGTGGGCGACTAC-3'<br>Reverse 5'-GCAGGGCGGGCATAAAGACG-3'
Cp19	CG6524	Chorion protein	Forward 5'-GGAGCCGAGGGTCAGCAGCGTTAT-3'<br>Reverse 5'-TGGAGCAGCAGGGCGGATGG-3'
**Unknown**	CG17571	Trypsin-like cysteine/serine peptidase	Forward 5'-CTCCTCCCCCGACACCCTTCA-3'<br>Reverse 5'-GCTGGCGACATCGGCATAGACG-3'
Lsd-1	CG10374	Perilipin domain, lipid mobilization	Forward 5'-TGAGCCGGCGACAGCAACAGT-3'<br>Reverse 5'-CGTAGGCGGCCGAAATGGTG-3'
